# Cost-effectiveness analysis of biologic sequential treatments for moderate-to-severe psoriasis: A Malaysian healthcare system perspective

**DOI:** 10.1371/journal.pone.0307234

**Published:** 2024-09-06

**Authors:** Nor Azmaniza Azizam, Mofakhar Hussain, Eric Nauenberg, Wei Chern Ang, Amirah Azzeri, Jacob Smith

**Affiliations:** 1 Institute of Health Policy, Management and Evaluation, University of Toronto, Toronto, ON, Canada; 2 Faculty of Business and Management, Universiti Teknologi MARA Puncak Alam Campus, Selangor, Malaysia; 3 Clinical Research Centre, Ministry of Health Malaysia, Hospital Tuanku Fauziah, Kangar, Malaysia; 4 Department of Pharmacy, Hospital Tuanku Fauziah, Ministry of Health Malaysia, Kangar, Malaysia; 5 Faculty of Medicine and Health Sciences, Department of Primary Care, Public Health Unit, Universiti Sains Islam Malaysia, Nilai, Malaysia; The First Hospital of Jilin University, CHINA

## Abstract

**Objective:**

In Malaysia, there is now a dearth of recommendations pertaining to the priority of biologic treatments for the effective management of psoriasis, given the multitude of available therapeutic alternatives. Present analysis reports results of a cost-effectiveness model that determines the most optimal arrangement of biologic treatments, with a particular focus of adding biosimilars to the existing treatment pathway for psoriasis in Malaysia.

**Methods:**

A Markov model was developed to compare the cost effectiveness of various biologic sequential treatments in a hypothetical cohort of moderate to severe psoriasis patient in Malaysia over a lifetime horizon. The model simulated the progression of patients through three lines of active biologic therapy, before transitioning to best supportive care. Costs and effects were discounted annually at a rate of 3%.

**Results:**

First line secukinumab has produced lowest incremental cost effectiveness ratios (ICERs) when compared to first line systemic [ICERs value; US$152,474 (first set analysis) and US$110,572 (second set analysis)] and first line phototherapy [ICERs value; US$147,057 (first set analysis) and US$107,616 (second set analysis)]. However, these values were slightly higher than the Malaysian based threshold of three times gross domestic product per capita, US$104,337. A 40% reduction in the unit costs of reference biologics renders most of the evaluated treatment sequences cost-effective.

**Conclusion:**

Adding biosimilar to the current treatment sequence could achieve cost savings ranging from 4.3% to 10.8% without significant loss of effectiveness. Given the significant impact of comorbidities and the resulting decline in quality of life among individuals with psoriasis, it may be justifiable to establish a threshold of up to US$184,000 per quality-adjusted life year (QALY) for the provision of therapies in the context of Malaysia.

## Introduction

Psoriasis is an immune-mediated, inflammatory diseases that affects skin and is associated with quality of life impairment [1]. Psoriasis is recently identified as a systemic condition, with psychosocial, metabolic, arthritic, and cardiovascular comorbidities [2–4]. The prevalence of psoriasis in the general population has been reported to vary from 0.11% in East Asia to 1.58% in Australasia, and 1.52% in Western Europe [5]. Psoriasis prevalence in Malaysia would be comparable to that in other Asian countries ranging from 0.2% to 0.5% [6,7]. 21%-30% of those Psoriasis have moderate to severe disease [8]. Psoriasis often impacts individuals within the working-age population [9] and has been shown to diminish the work capacity and lead to premature retirement [2,10]. Research findings suggest that individuals diagnosed with moderate to severe psoriasis have 15–20% decline in their capacity to engage in work-related activities [11–13].

The choice of therapy for psoriasis is contingent upon the clinical evaluation of its severity. The aforementioned measures include the Psoriasis Area and Severity Index (PASI), Physician Global Assessment (PGA), and Dermatology Life Quality Index (DLQI). According to the Malaysian Clinical Practise Guideline (CPG), the first treatment approach for mild psoriasis involves the use of topical medication, which has shown efficacy in managing mild condition. The management of moderate to severe psoriasis is more difficult, and it may require systemic therapy with non-biologic or biologic drugs rather than phototherapy [14,15]. In Malaysia, numerous biologics, including inhibitors targeting anti-tumour necrosis factor (TNF), anti-interleukin (IL) 12/23, IL-17, and IL-23, have been approved and are now accessible for the treatment of moderate to severe psoriasis. According to data from the Malaysian Psoriasis Registry (MPR) spanning from 2007 to 2019, a total of total 23,803 psoriasis patients were notified to the registry. Of this, 203 or 1.1% have received biologic therapy for their psoriasis. Among the biologics prescribed, ustekinumab accounted for 35.1% of the cases, followed by adalimumab at 26.4% and secukinumab at 20.2% [16]. A recent network meta-analysis (NMA) based on randomized controlled trials (RCTs) revealed that secukinumab, consistently exhibited superior efficacy compared to ustekinumab, and adalimumab [17]. However, the high cost associated with these treatments poses a barrier to patient accessibility. Biological therapies have become a progressively significant aspect of pharmaceutical spending, mostly attributed to their effectiveness in addressing intricate complex diseases. Biologics account for 35% of pharmaceutical expenditure in Europe at the listed rates, and have seen a compound annual growth rate (CAGR) of 11.3% during the previous five-year period [18]. A cost-effectiveness analysis conducted on psoriasis treatments in Malaysia revealed that cost of biologics accounted for over 40% of the total direct costs (such as monitoring services and radiology examination) with an estimated annual cost per patient of US$37,304.09 (adjusted for inflation to 2022) [19]. The utilization of biosimilars has emerged as one of the potential solutions to the problem of the high cost of biologics [20]. In 2010, the Malaysian’s National Pharmaceutical Regulatory Agency (NPRA), has approved the first biosimilar medicine containing somatropin; since then, the country has seen a surge in the number of biosimilars, which reached 38 as of October 2022 and adalimumab biosimilar (brand name amgevita) was approved to be used in treating psoriasis [20]. There is scarce information available on utilization patterns of biosimilars in Malaysia [21].

Advancements in biologic treatments have brought about a shift in the approach to managing moderate to severe psoriasis, leading to improved effectiveness and enhanced quality of life for patients. Despite of this, there is a rising demand for switching to alternative biologics in clinical settings due to long term efficacy loss and adverse effects [22]. However, there is a lack of clear guidance regarding the prioritization of biologic therapies in Malaysia. Consequently, it is reasonable to assume that may be implementing treatment sequences that are suboptimal in terms of cost-effectiveness. With the continuous development of new mode-of-action biologics and biosimilars, the decision-making process for selecting the appropriate treatment sequence becomes more challenging [23]. Adalimumab biosimilar (brand name Amgevita) has been approved for psoriasis in Malaysia [24], however, little is known about its utilization in the management for moderate to severe psoriasis in Malaysia. Therefore, this study aimed to develop a cost-effectiveness model to determine the optimal sequence of biologic therapies, with a particular focus of adding biosimilar to the current treatment pathway for moderate to severe psoriasis in Malaysia.

## Methods

A time-dependent cohort state-transition model was developed to evaluate the cost-effectiveness of treatment sequences for moderate to severe psoriasis in Malaysia. The model structure adapted from previous study done by Sun et al. [25] which evaluated the three lines of biologic sequencing treatments for psoriasis. Annual discounting of 3% used for costs and health effects following the Pharmacoeconomic Guideline for Malaysia [26]. The results of the analysis were summarized as an incremental cost-effectiveness ratio (ICER). To conclude the cost-effectiveness in this analysis, the ICER was compared with a national cost-effectiveness threshold (CET). A treatment sequence is deemed cost-effective if it falls below the suggested threshold of three times the Gross Domestic Product (GDP) per capita, US$104,337.19 [27–29].

### Model structure

A Markov model was developed to reflect the disease progression of psoriasis and the treatment effect of sequences of biologic treatments compared to first line, phototherapy (PHOTO) and systemic (SYS) treatments. PHOTO includes ultraviolet B (UVB) phototherapy, psoralen and ultraviolet A (UVA) and SYS includes acitretin, cyclosporin, sulfasalazine and methotrexate [19,30]. In Malaysia, SYS and PHOTO are the first line treatment for moderate to severe psoriasis. Biologic therapy should be used only in severe psoriasis (PASI score >20, DLQI >20 or BSA >30%) in situations where treatment has failed or is contraindicated, or the patient is intolerant to nonbiologic. Patients who do not achieve a satisfactory response with the first line biologic treatment, it is recommended to consider switching to other biologics or combining them with methotrexate or NBUVB therapy [15]. According to the Malaysian Psoriasis Registry (MPR) data from 2007 to 2019, only 6.1% of patients were treated with systemics, phototherapy, and biologics. Of these, the majority were receiving systemics (19.2%), followed by phototherapy (2.6%), and biologics were used in 1.1% of patients. Additionally, among all available biologics, ustekinumab and adalimumab were used in 35.1% and 26.4% of patients, respectively [16]. Hence, they were selected as comparators in this study. All biologics were evaluated based on approved licensed schedule [17]. Table 1 showed the biologics considered and approved dosing schedules for cost effectiveness analysis.

**Table 1:** Dosing schedules.

Treatment	Dosing schedule
Secukinumab (SEC)	300 mg at weeks 0, 1, 2, 3, and 4, then every 4 weeks
Ustekinumab (UST):	45 mg at weeks 0 and 4, then every 12 weeks
Adalimumab (ADA):	80 mg at week 0, then 40 mg every 2 weeks
Adalimumab biosimilar (ADA BS):	80 mg at week 0, then 40 mg every 2 weeks

**Fig 1:**
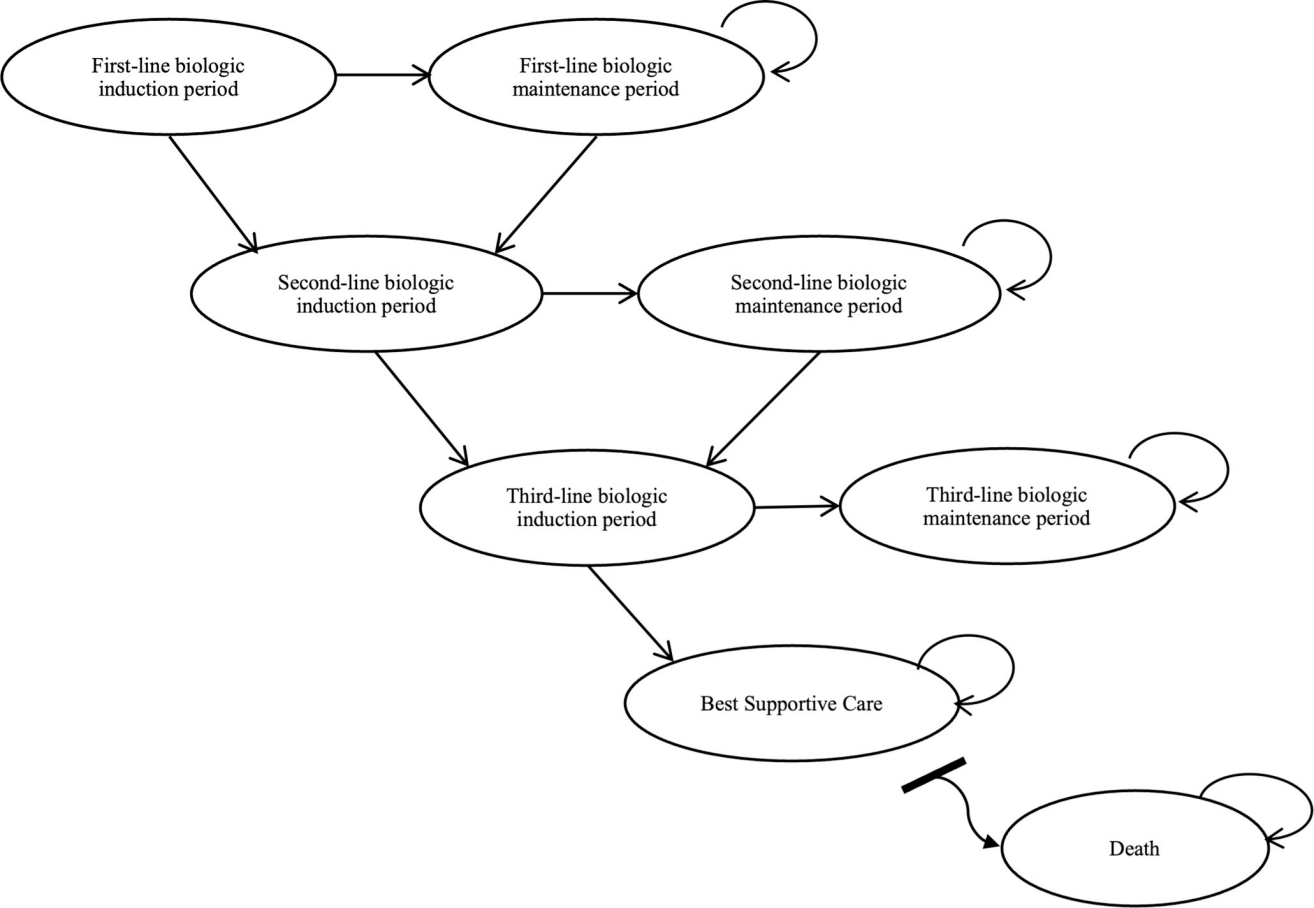
Markov model for moderate to severe psoriasis.

A lifetime horizon was adopted to comprehensively capture meaningful variations in costs and health impacts of treatments as observed in prior research [31–33]. The analysis was from a hypothetical cohort of psoriasis patients aged ≥35 years who had an indication to start biologic therapy in Malaysia. The cohort starting age in the model was set to 35 years old to align with the average age of onset among adult patients in Malaysia, as reported in the MPR from 2013 to 2019 [16]. The Malaysian guideline for the treatment of psoriasis recommends biologic therapy only for patients with severe psoriasis (psoriasis area severity index, PASI score >20, dermatology life quality index, DLQI >20, or body surface area, BSA >30%) when non-biologic therapies (SYS or PHOTO) has failed, is contraindicated, or the patient is intolerant [15,34]. Each Markov cycle corresponds to 16-weeks and included four health states: treatment induction, maintenance, best supportive care (BSC), and death. Treatment responses were based on PASI response at the end of induction period, consistent with previous economic evaluation studies [17,35,36]. Patients who attained PASI75 (75% improvement from baseline) at the end of the induction period continued treatment to the 16-weeks maintenance state and remain in the same health state unless death occurs within this state. There is no evidence indicating that drug reaction improves after the initiation phase [37]. Patients who did not achieve PASI75 at the end of the induction period, discontinue first-line therapy. Provided that there is a lack of evidence regarding switching practice in Malaysia, patients discontinued from their first biologic treatment, they are assumed to transition through two additional lines of biologic prior to BSC, which patients were assumed to remain on until their death. Patients could transition to death from any health state (Fig 1). Patients who failed to achieve PASI75 were, on average, assumed to fail halfway through a 16-week cycle. Within-cycle correction was used to apply this assumption.

In these analyses, the number of permutations was narrowed by focusing on the most prescribed biologics. Currently, it is understood that UST, ADA, and SEC are the most frequently prescribed biologics based on the available data [16] in Malaysia. Hence, these originator biologics made up the first analysis set. Second set analysis was conducted to explore the impact of adding ADA BS into each of these sequences (Table 2).

**Table 2:** Treatment sequence assumptions.

Analysis	Sequence
Comparators	SYS →UST→ADA→BSC PHOTO →UST→ADA→BSC
First set analysis. Biologic reference sequences	UST →ADA→SEC→BSC UST →SEC→ADA→BSC SEC→UST→ADA→BSC SEC→ADA→UST→BSC ADA→UST→SEC→BSC ADA→SEC→UST→BSC
Second set analysis. Biosimilar treatment pathways	ADA BS →UST→ SEC→BSC ADA BS→SEC→UST→BSC UST→ ADA BS→SEC→BSC SEC→ ADA BS → UST →BSC UST→SEC →ADA BS→BSC SEC→ UST→ ADA BS→BSC

#### Model input–efficacy

PASI75 is considered the primary endpoint in the base case study as seen in various economic evaluation studies to evaluate the efficacy of biologics as psoriasis therapies [25,37,38]. In the absence of PASI response rates data from MPR, PASI75 was used to inform the model for biologics were sourced from network meta-analysis (NMA), which synthesized data from 60 randomized controlled trials (RCTs) of biologics in short term period (12–16 weeks) [17]. The short term PASI75 response rate for PHOTO and SYS (16 weeks) were taken from meta-analysis, which synthesized data from mix treatment comparison based on RCTs [39,40]. Due to unavailable credible intervals (CrI) reported in the meta-analysis by Liu et al. [39], a ±20% variation was applied to the probability of the PASI75 response rate for PHOTO, following a study on the economic evaluation of psoriasis treatments in Malaysia [19] and previous cost effectiveness analysis of phototherapy for psoriasis [41]. Similar efficacy rate was applied to ADA and its biosimilar. This supported by evidence demonstrating comparable efficacy, tolerability, and immunogenicity between ADA BS and its reference drug in patients with psoriasis [42] and rheumatoid arthritis [43,44]. All the inputs were used for the induction period transition probabilities (Table 3).

**Table 3:** Model input value.

Input	Baseline value	Deterministic sensitivity analysis range	Probabilistic sensitivity analysis distribution	Source
**Unit cost of drugs**				
SEC (per 150 mg)	3,555	2,844–4,266	Gamma (μ,σ)	[31]
UST (per 45 mg)	10,379	8,303–12,455	Gamma (μ,σ)	[31]
ADA (per 40 mg)	1,481	1,185–1,778	Gamma (μ,σ)	[31]
ADA BS (per 40 mg)	886	709–1,063	Gamma (μ,σ)	[45]
SYS	1,183	946–1,420	Gamma (μ,σ)	[19]
PHOTO	1,427	1,141–1,712	Gamma (μ,σ)	[19]
**Unit cost of monitoring tests**				
Biologics (SEC, UST, ADA, ADA BS)	168	134.18–201	Gamma (μ,σ)	[46]
SYS	299	239.37–359	Gamma (μ,σ)	[46]
PHOTO	185	147.73–222	Gamma (μ,σ)	[46]
Outpatient costs	71	57–86	Gamma (μ,σ)	[47]
**Utility inputs**				
**PASI75**				
Biologics (SEC, UST, ADA, ADA BS)	0.85	0.68–1.00	Beta (μ,σ)	[15,19,48]
SYS	0.89	0.71–1.00	Beta (μ,σ)	[15,19,48]
**PASI<75**				
Biologics (SEC, UST, ADA, ADA BS)	0.62	0.50–0.74	Beta (μ,σ)	[15,19,48]
SYS	0.64	0.51–0.77	Beta (μ,σ)	[15,19,48]
BSC	0.11	0.09–0.13	Beta (μ,σ)	[49]
**Probability inputs**				
**PASI75**				
SEC	0.85	0.83–0.88	Beta (μ,σ)	[17]
UST	0.70	0.65–0.74	Beta (μ,σ)	[17]
ADA	0.70	0.66–0.73	Beta (μ,σ)	[17]
ADA BS	0.70	0.66–0.73	Beta (μ,σ)	[17]
SYS	0.42	0.27–0.54	Beta (μ,σ)	[50]
PHOTO*	0.32	0.18–0.26	Beta (μ,σ)	[39]
Discount rate, %	0.03	0.00–0.01	Gamma (μ,σ)	[26,39]
**All cause annual discontinuation risk**				
SEC	0.22	0.18–0.26	Beta (μ,σ)	[31]
UST	0.20	0.16–0.24	Beta (μ,σ)	[31]
ADA	0.21	0.17–0.25	Beta (μ,σ)	[31]
ADA BS	0.21	0.17–0.25	Beta (μ,σ)	[31]
SYS	0.38	0.30–0.46	Beta (μ,σ)	[31]
PHOTO	0.49	0.39–0.59	Beta (μ,σ)	[31]

#### Model input—Cost input

The model includes only direct costs which include drug acquisition costs, administration costs, and cost of monitoring tests. The cost of adverse events is excluded from the analysis because the biologics assessed reported to have similar adverse events [51]. The unit price of reference biologics was adopted from Saeki et al. [31] and the unit price for ADA BS was obtained from Ontario Drug Benefit Formulary [52]. All costs were adjusted to Malaysian Ringgit (RM) using the Central Bank of Malaysia exchange rate [53] for the year 2022 (JPY100 = RM3.62) and (1RM = CAD$3.30) (Table 3). All the costs in RM were then converted to United States dollars (USD) using Purchasing Power Parity (PPP) for the year of 2022 (1.58  =  1 USD) [54]. Drug administrations were considered as provider-administered injections for SEC and UST corresponding to approved dosing schedules for UST and SEC [17] and applied once for ADA and its biosimilar. Total costs of administration were calculated by multiplying the cost of outpatient [47] by the frequency of drug administrations (Table 3). In this analysis, 2 sessions per week of narrowband UVB was used as proxy to calculate the total cost of administration for phototherapy [15]. Monitoring tests included initial screening tests and during treatment to monitor for common biologic side effects [8,55]. The cost of monitoring tests included the costs of diagnostic tests associated with all treatments, and routine outpatient visits (Table 3). Cost of drugs and monitoring tests associated with systemics, and phototherapy were obtained from previous cost effectiveness analysis of psoriasis treatment modalities in Malaysia [11]. BSC costs incorporated costs associated with topical treatments, outpatient, and inpatient managements [19,56].

#### Utility input

QALYs were accumulated based on whether the patient achieved PASI75. The number of QALY gained for each health state was estimated based on a preference-based utility scoring algorithm, using time trade off, provided by Matza et al. [48]. Current local recommendations are that systemic and phototherapy are first line treatment for moderate to severe psoriasis (BSA 10% to 30% or PASI 10 to <20, DLQI 6 to <18) and biologic therapy should be used only in severe psoriasis (PASI score >20, DLQI >20 or BSA >30%) in situations where treatment has failed or is contraindicated or the patient is intolerant to non-biologics [15]. Previous analysis demonstrated that the mean baseline PASI score for moderate to severe psoriasis in Malaysia was PASI16.02±10 [19]. According to Matza et al. [48], the patient’s pre-treatment baseline was assumed to be PASI16.5 and PASI20.3, the lowest value that was eligible for a starting baseline PASI score for moderate and severe psoriasis in Malaysia. Utility associated with BSC (topical treatment) was adopted from a cost utility study of psoriasis treatments [49] (Table 3).

#### Discontinuation risk

Treatment specific discontinuation risk was applied to the patients in a maintenance state to consider the loss of efficacy or tolerability over time and to enable the demonstration of any differences between biologics. The annual probability of discontinuation for biologics, SYS and PHOTO were adopted from previous analyses [31,39,49,57] and were converted to 16 weeks to represent the discontinuation during the induction period. The model assumed biosimilar treatment had equivalent discontinuation probability to its reference product (Table 3).

#### Mortality input

Age-dependent all-cause mortality was based on the abridged life table for Malaysian [58]. At every cycle of the model, the patients can die of natural causes. According to the MPR [16], the mean of onset for adults’ psoriasis was 35 years old. Therefore, the starting age in the model was 35 years and the mortality rate in the model was based on mortality for both male and female. The model applies an elevated all-cause mortality risk in severe psoriasis (Relative Risk;1.52) [59] to the general Malaysian life table. Annual mortality rates were converted to 16-week probabilities.

#### Sensitivity analysis

Sensitivity analyses were performed to address the inherent uncertainties of economic modelling that relies on data gathered from multiple sources, as well as the assumptions and inputs where data is deficient. In one-way sensitivity analyses, the unit cost of drugs, cost of outpatient, costs of BSC, probability of PASI75, utility values, discontinuation risk were varied. For this model, upper and lower confidence intervals were used where available. In unavailable, input parameters were varied by ±20%, and this was consistent with other economic evaluation of biologics for psoriasis [31,60,61]. Annual discount rate was varied 1% and 5% to evaluate their influence on the ICER. Tornado diagrams were used to present the results of the one-way deterministic sensitivity analysis (OWSA). In order to determine the extent to which unit price originator biologics reductions would make the reference biologics treatment sequences cost-effective in the Malaysian context, a scenario analysis was conducted. Four scenarios were considered, with reductions of 20%, 30%, 40%, and 50% in the biologics reference unit cost. This scenario was developed by taking into account of the variation in the sensitivity analysis for biologics in various settings such as in the UK, Japan [31,60,61] and amount of reduction in biologic unit prices required to render biologics cost-effective and fall below the cost-effectiveness threshold in Brazil [36]. A probabilistic sensitivity analysis (PSA) was conducted to account for uncertainty across all model parameters simultaneously. Probabilistic ICERs were obtained from averaging the total costs and QALYs accrued after sampling from distributions of the input parameters in 10,000 iterations. A gamma distribution was assigned for costs and the beta distribution was considered for probability and utility variables. For all variables, a standard error of 10% of the average value was considered. A cost-effectiveness acceptability curve (CEAC) showed the PSA results. To complement PSA results, expected net benefit loss from each strategy was quantified over a range of WTP thresholds and presented via expected loss curves (ELCs). The analyses were conducted using R software follows a conceptual algorithm for cost effectiveness model implementation in R developed Alarid-Escudero et al. [62]. The model source codes was made available at https://github.com/norazmaniza/CEA_sequences.

#### Ethics statement

All inputs used to build the model were extracted from open, published and publicly available sources. Input costs were derived from previous economic evaluation analyses. Information on efficacy/effectiveness of treatments were sourced from a network meta-analysis based on randomized controlled trials. Some information used to build the treatment sequences were based on the published and publicly available report from Malaysian Psoriasis Registry. No intervention or procedure was conducted on patients nor was data extracted data from medical records. Therefore, no ethical review was required.

#### Base case

**Fig 2:**
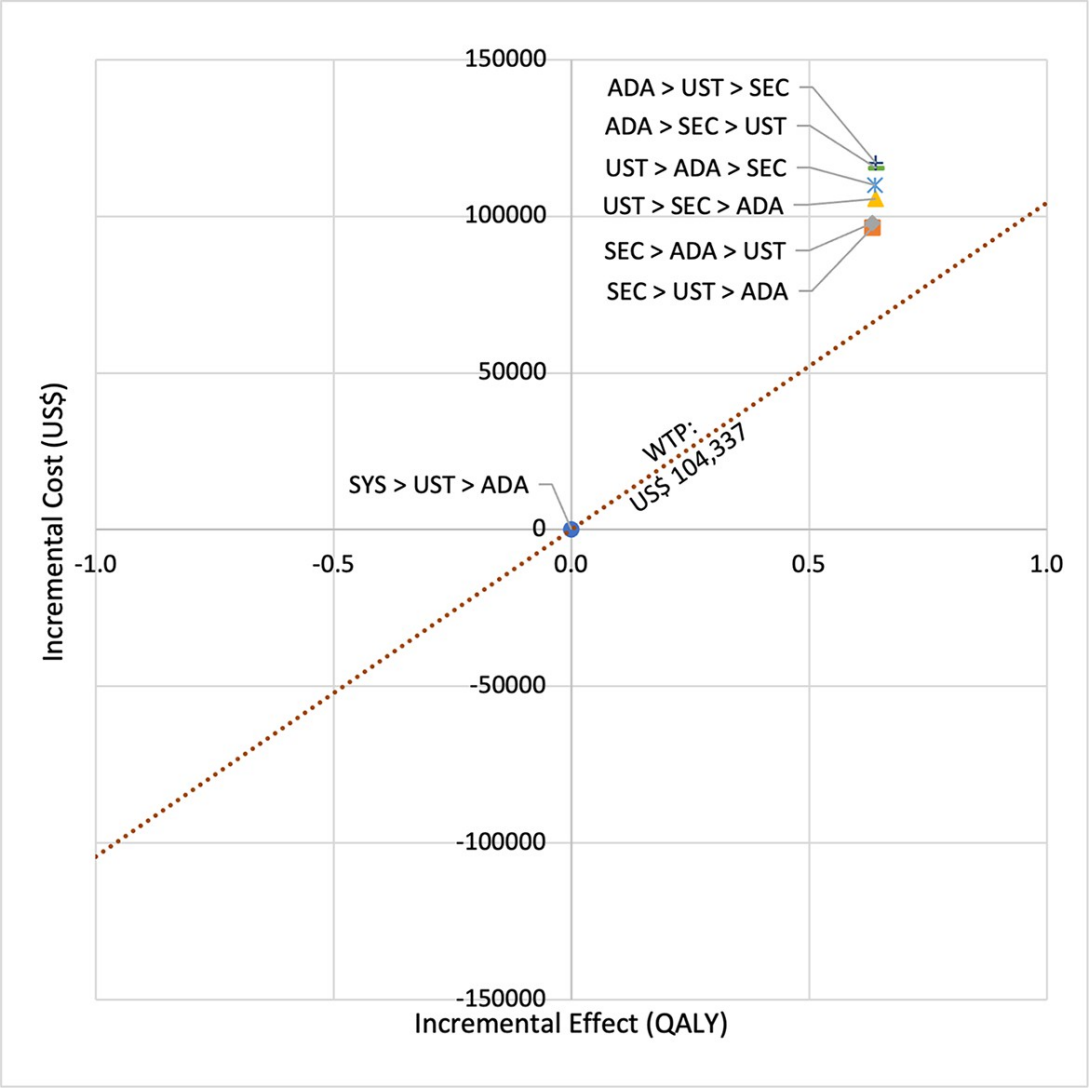
Cost effectiveness analysis plane (reference biologic sequences versus first line SYS).

**Fig 3:**
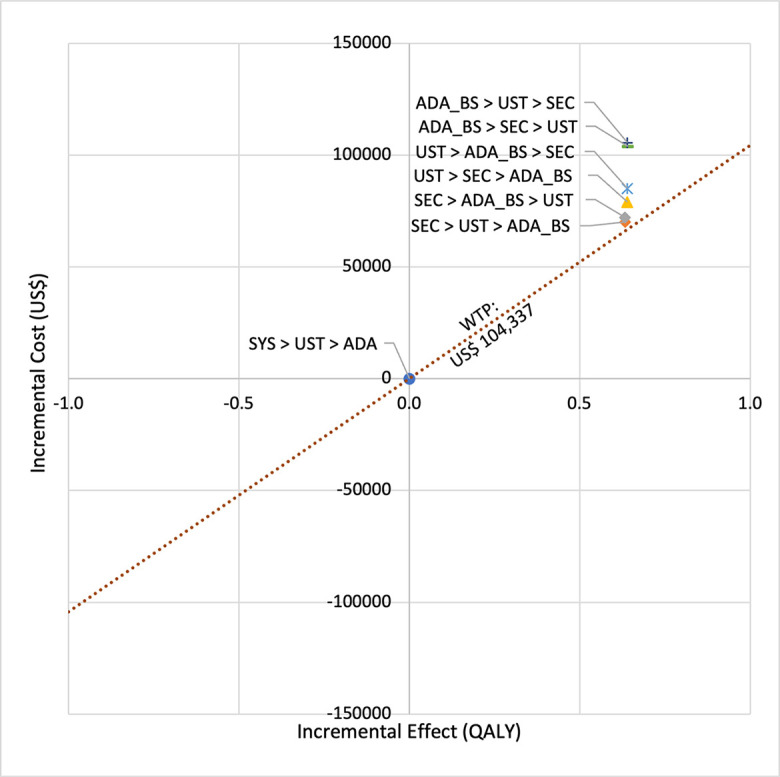
Cost effectiveness analysis plane (biosimilar sequences versus first line SYS).

**Fig 4:**
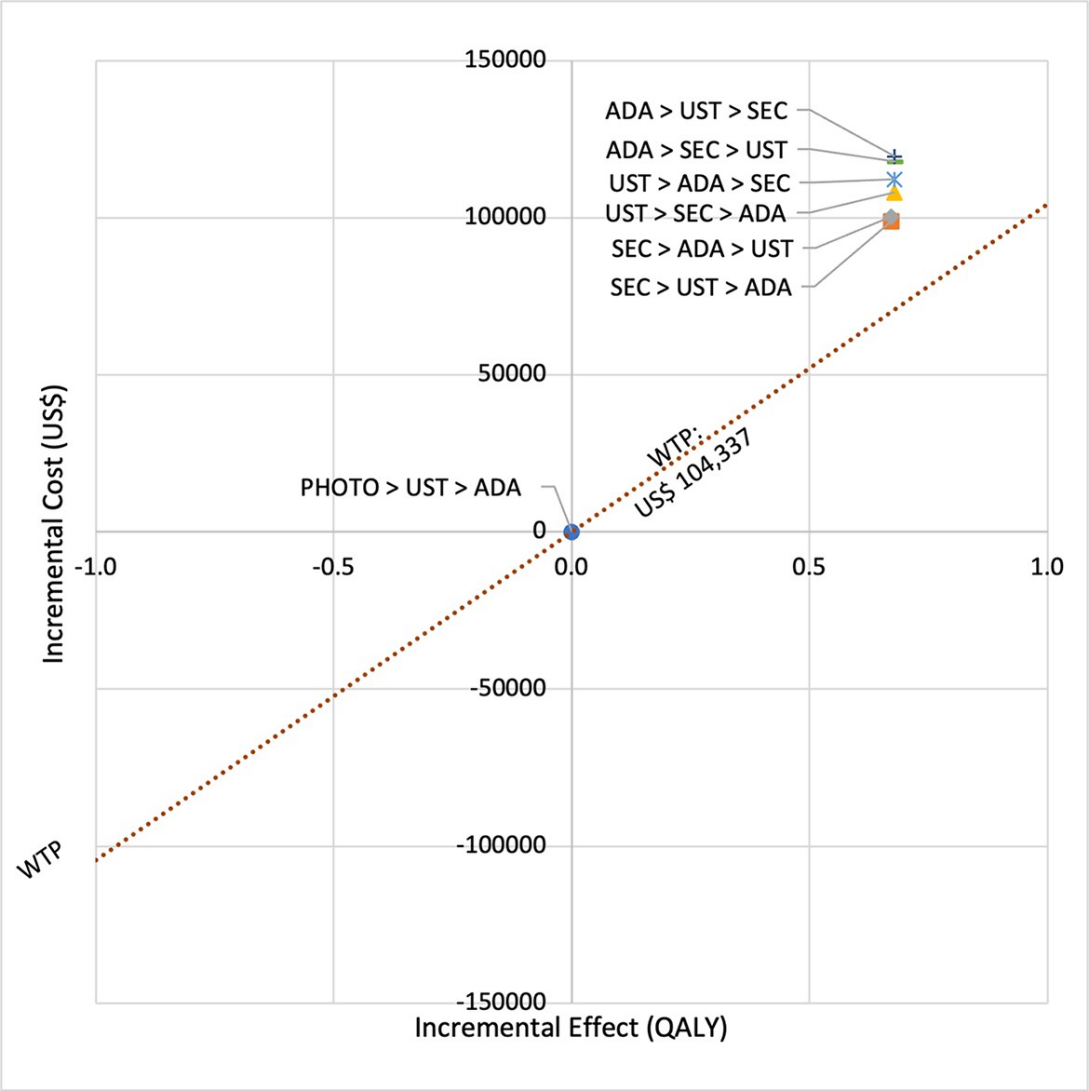
Cost effectiveness analysis plane (reference biologic sequences versus first line PHOTO).

**Fig 5:**
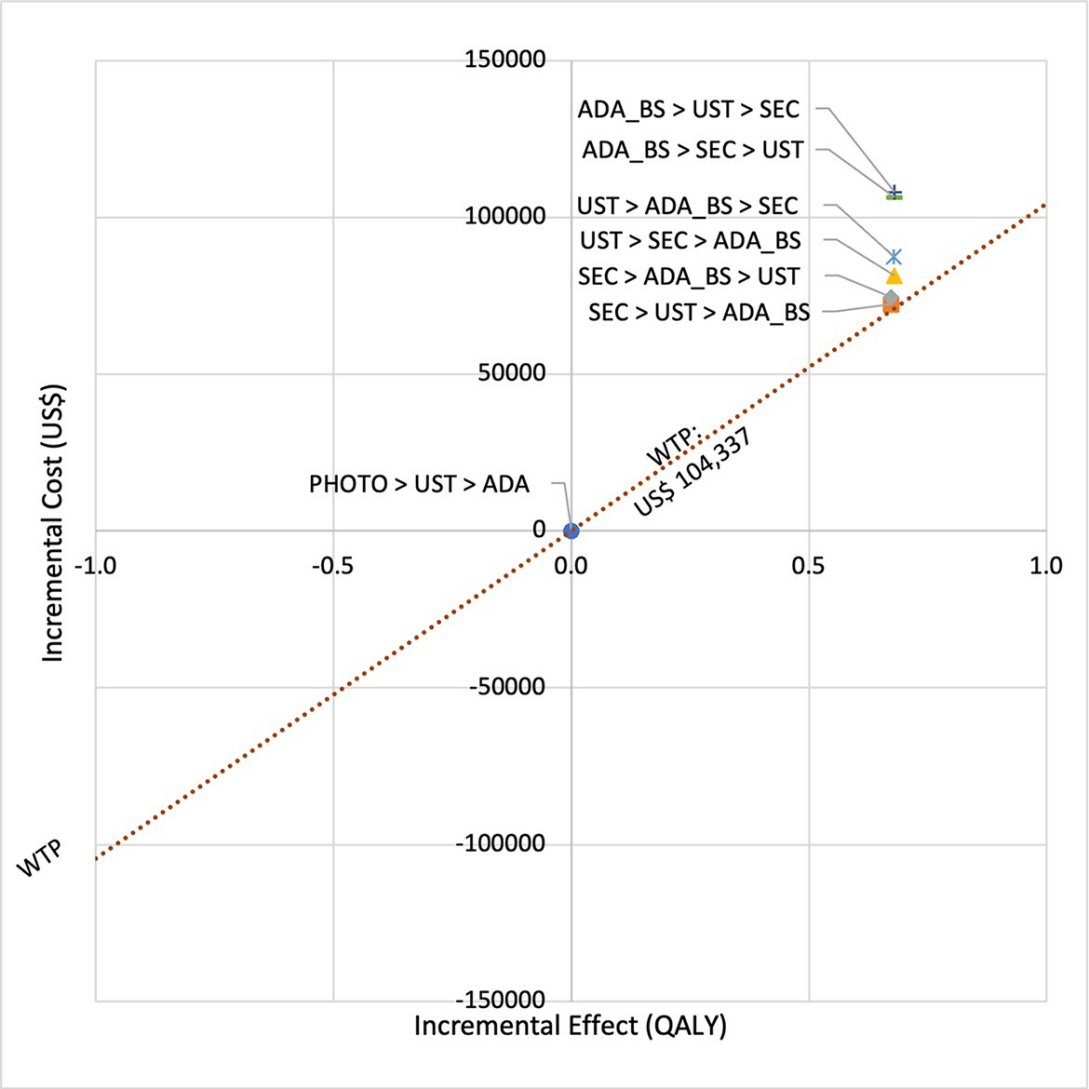
Cost effectiveness analysis plane (biosimilar sequences versus first line PHOTO).

**Fig 6:**
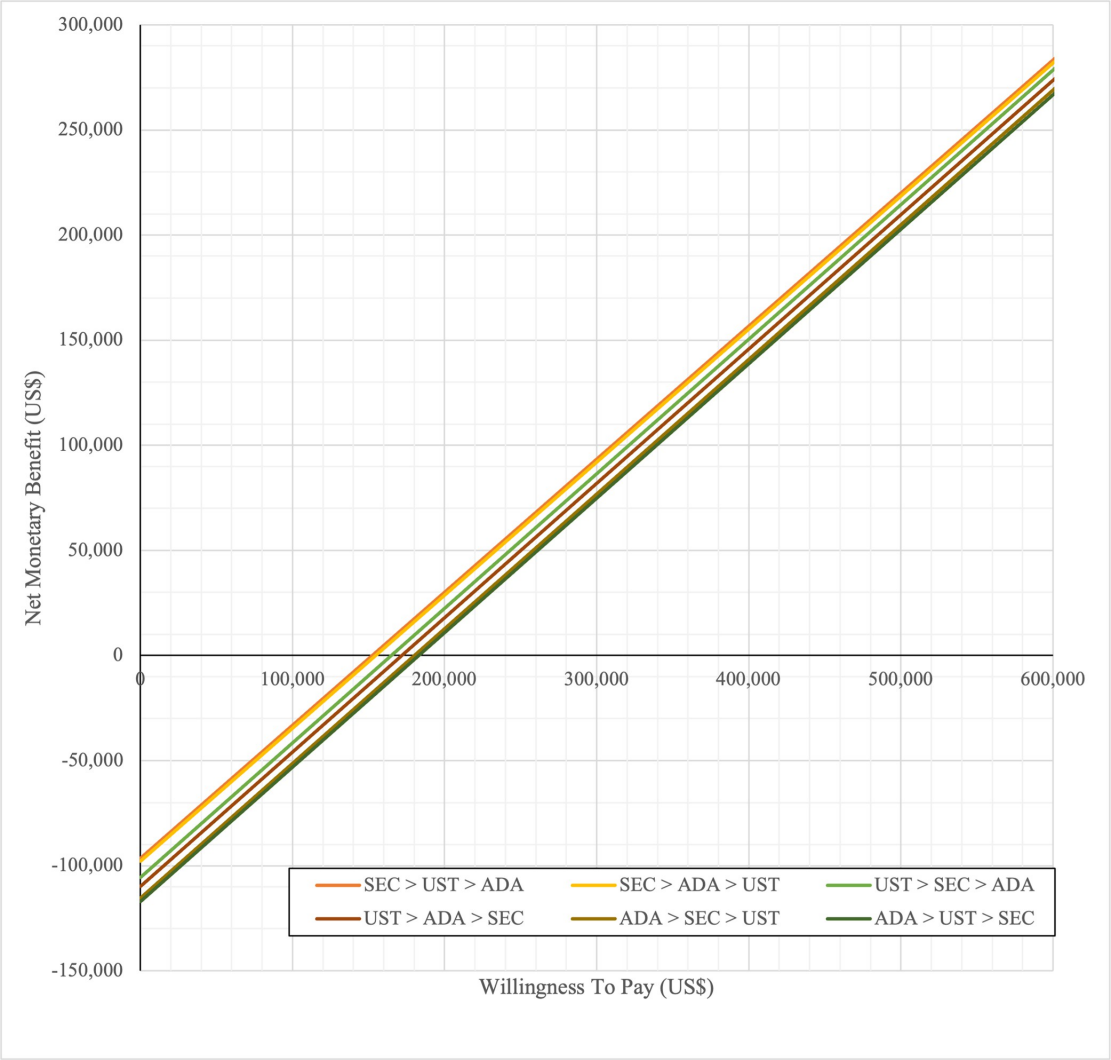
Net monetary benefit (reference biologic sequences versus first line SYS).

**Fig 7:**
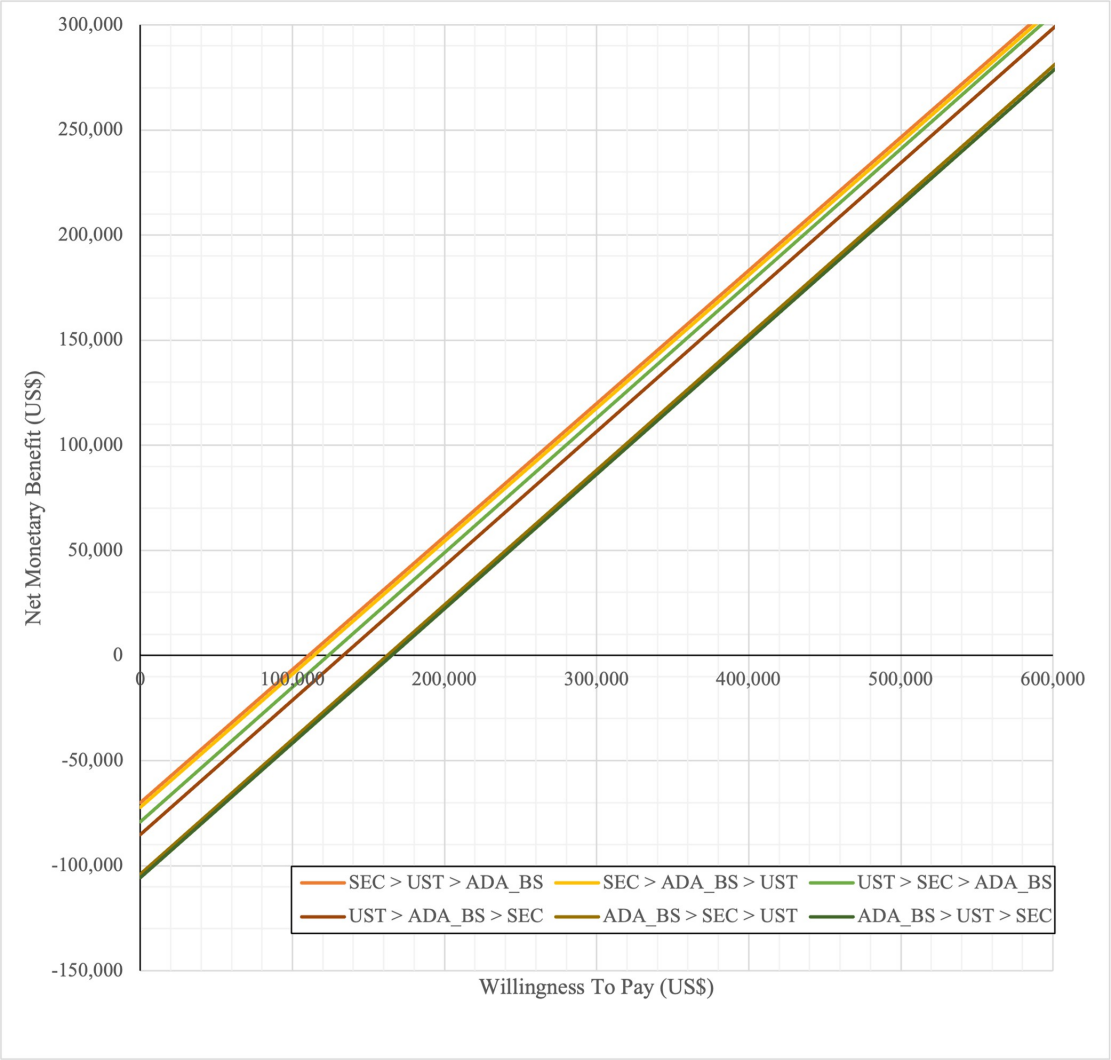
Net monetary benefit (biosimilar sequences versus first line SYS).

**Fig 8:**
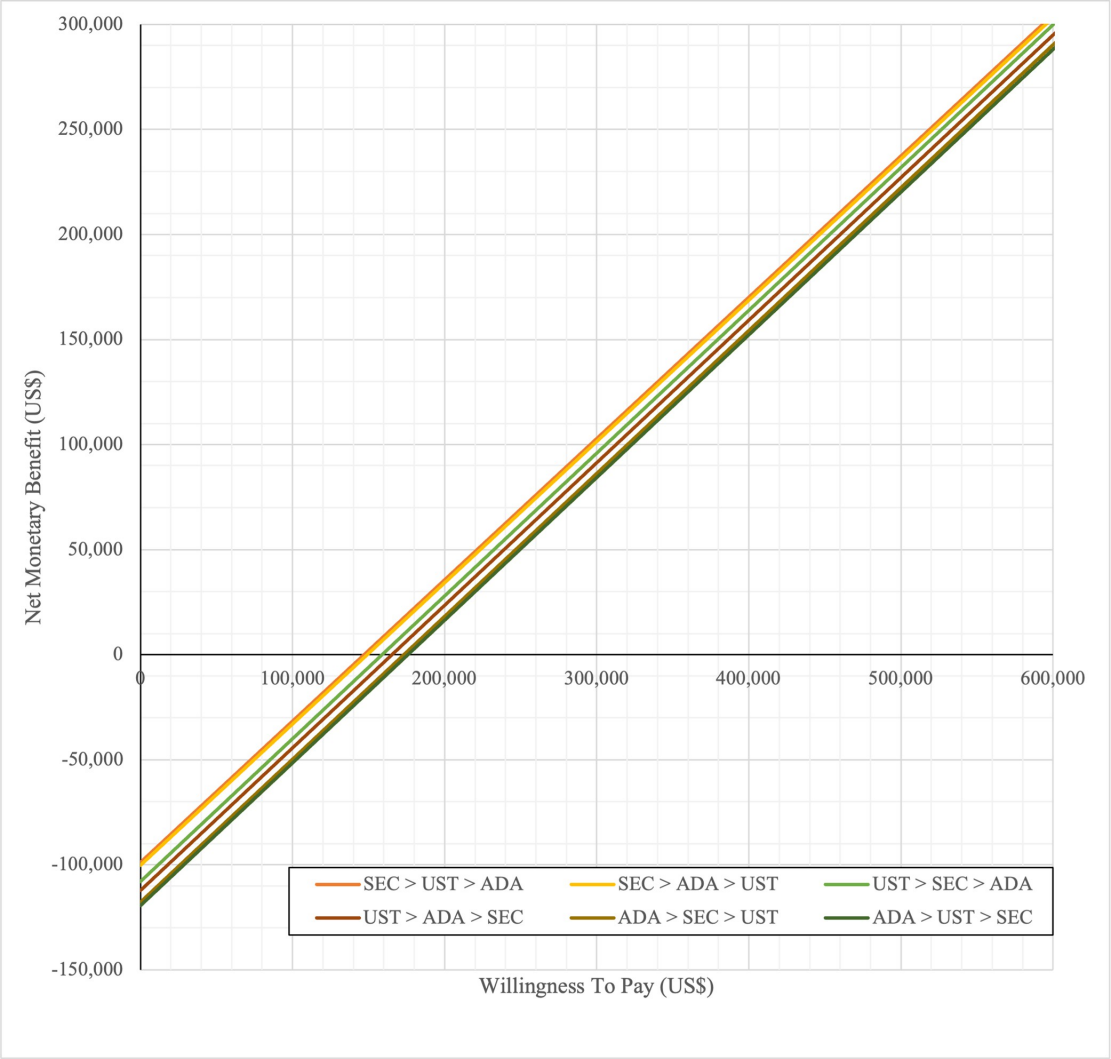
Net monetary benefit (reference biologic sequences versus first line PHOTO).

**Fig 9:**
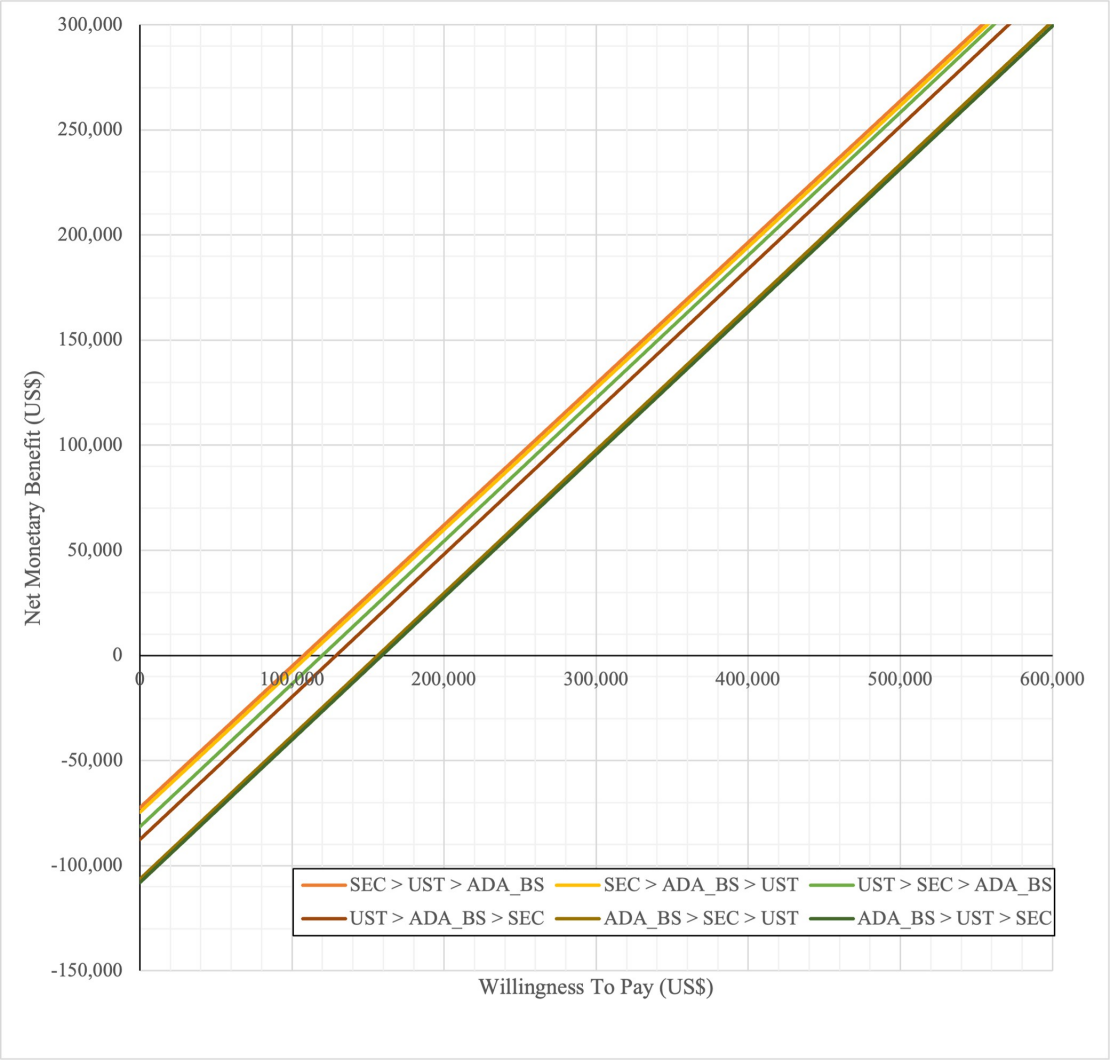
Net monetary benefit (biosimilar sequences versus first line PHOTO).

In first set analysis, SEC→UST→ADA produced the lowest ICER, US$152,474.11/QALY and US$147,057/QALY when compared to SYS→UST→ADA and PHOTO→UST→ADA, respectively. Second analysis set was undertaken in which the impact of adding biosimilar ADA into each of these sequences was examined. Results showed that the replacing reference drug with its biosimilar has reduced the cost with no significant loss of QALYs. SEC→UST→ADA BS was the most cost effective, with the lowest ICER value of US$110,572/QALY and US$107,616/QALY when compared to SYS→UST→ADA and PHOTO→UST→ADA, respectively. Considering the national based CET of US$104,337/QALY, none of these treatment sequences were cost effective in Malaysian setting (Tables 4 and 5) (Figs 2–5). A positive Net Monetary Benefit (NMB) indicates that the health benefits of a treatment exceed its costs. The positive NMB for SEC-UST-ADA appears at a WTP of at least $152,000 per QALY, which is higher than its biosimilar version at $110,600 per QALY, when compared to first-line SYS. Meanwhile, the positive NMB for SEC-UST-ADA BS appears at a WTP of at least $147,100 per QALY, higher than its biosimilar version at $107,700 per QALY when compared to first-line PHOTO (Figs 6–9).

**Table 4:** Incremental cost effectiveness analysis: Biosimilars and reference sequences vs first line SYS therapy.

Sequence	Total costs, US$	Total QALYs	ICER (US$/QALY)		
**First analysis set; reference biologic**					
**SYS→UST→ADA**	**149,721**	**3.30**	**-**		
SEC→UST→ADA	246,232	3.93	152,474		
SEC→ADA→UST	247,623	3.93	154,685		
UST→SEC→ADA	255,344	3.94	164,987		
UST→ADA→SEC	259,601	3.94	171,913		
ADA→SEC→UST	265,138	3.94	180,148		
ADA→UST→SEC	266,810	3.94	183,036		
**Second analysis set; adding ADA biosimilar**	**Total costs, US$**	**Total QALYs**	**ICER (US$/QALY)**	**Cost difference (US$)**	**Cost saving (%)**
SYS→UST→ADA	149,721	3.30	-		
SEC→UST→ADA BS	219,710	3.93	110,572	26,522	10.8
SEC→ADA BS→UST	221,933	3.93	114,096	25,690	10.4
UST→SEC→ADA BS	228,822	3.94	123,558	26,522	10.4
UST-ADA_BS-SEC	234,893	3.94	133,255	24,708	9.5
ADA BS→SEC→UST	253,718	3.94	162,323	11,420	4.3
ADA BS→UST→SEC	255,390	3.94	165,184	11,420	4.3

**Table 5:** Incremental cost effectiveness analysis; biosimilars and reference sequences vs first line PHOTO.

Sequence	Total costs, US$	Total QALYs	ICER (US$/QALY)		
**First analysis set; reference biologic**					
PHOTO→UST→ADA	147,343	3.30	-		
SEC→UST→ADA	246,232	3.93	147,057		
SEC→ADA→UST	247,623	3.93	149,138		
UST→SEC→ADA	255,344	3.94	158,901		
UST→ADA→SEC	259,601	3.94	165,415		
ADA→SEC→UST	265,138	3.94	173,187		
ADA→UST→SEC	266,810	3.94	175,896		
**Second analysis set; adding ADA biosimilar**	**Total costs, US$**	**Total QALYs**	**ICER (US$/QALY)**	**Cost difference (US$)**	**Cost saving (%)**
**PHOTO→UST→ADA**	147,343	3.30	-		
SEC→UST→ADA BS	219,710	3.93	107,616	26,522	10.8
SEC→ADA BS→UST	221,933	3.93	110,932	25,690	10.4
UST→SEC→ADA BS	228,822	3.94	119,879	26,522	10.4
UST→ADA BS→SEC	234,893	3.94	129,006	24,708	9.5
ADA BS→SEC→UST	253,718	3.94	156,396	11,420	4.3
ADA BS→UST→SEC	255,390	3.94	159,082	11,420	4.3

The additional analysis was conducted to examine the cost savings of biosimilars adoption. The cost difference was calculated by subtracting the cost of the originator from the cost of the biosimilar. Cost savings were calculated using the formula: (cost of originator–cost of biosimilar) / cost of originator × 100. The adoption of biosimilars can yield cost savings without significant loss efficacy when compared to first line SYS and first line PHOTO (comparators**).** The estimated cost savings associated with substituting ADA with its biosimilar could achieve cost savings ranging from 4.3% to 10.8% (US$11,420 to US$26,522) when compared to first line SYS and PHOTO comparators. (Tables 4 and 5).

#### Sensitivity analyses

**Fig 10:**
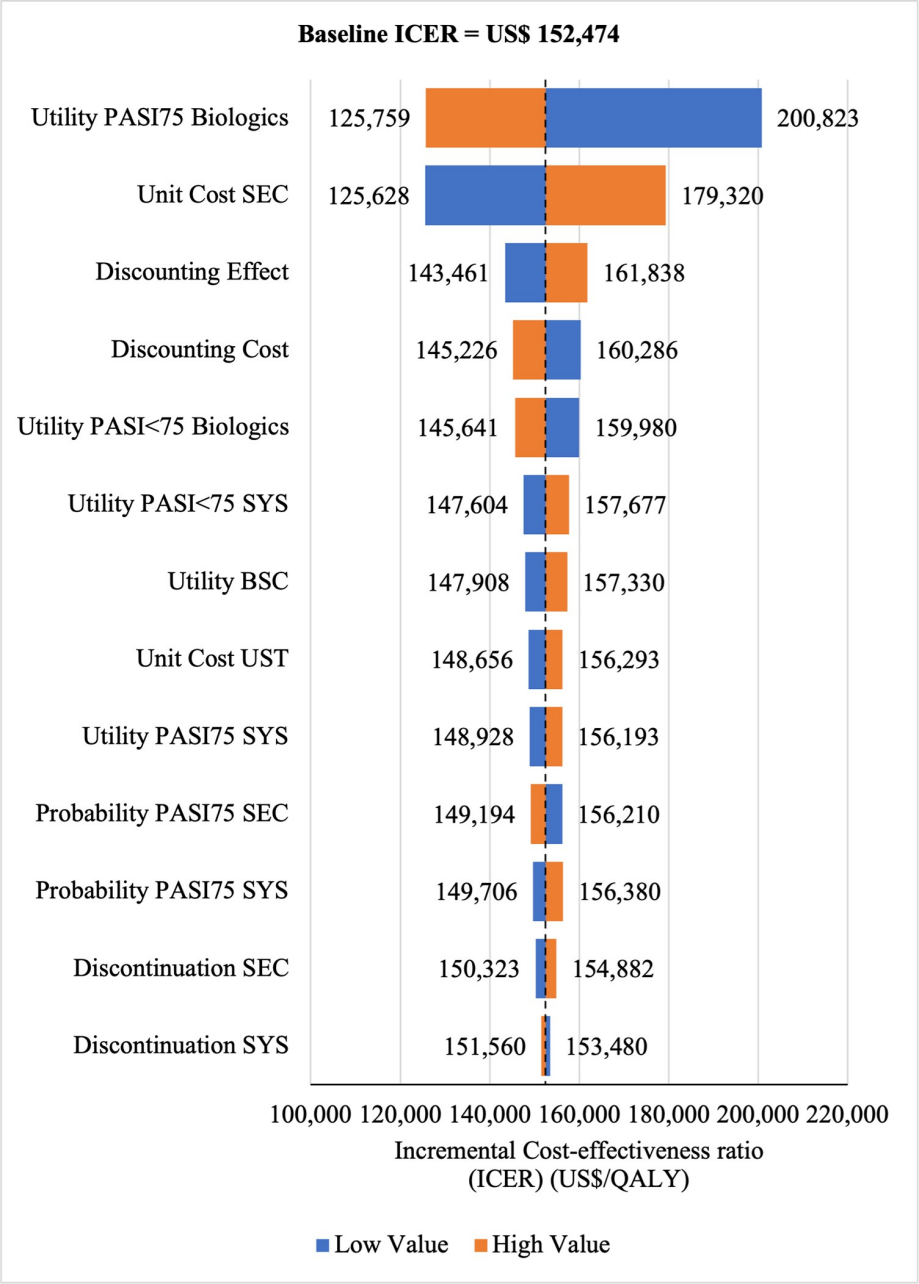
One way sensitivity analysis (reference biologic sequences versus first line SYS).

**Fig 11:**
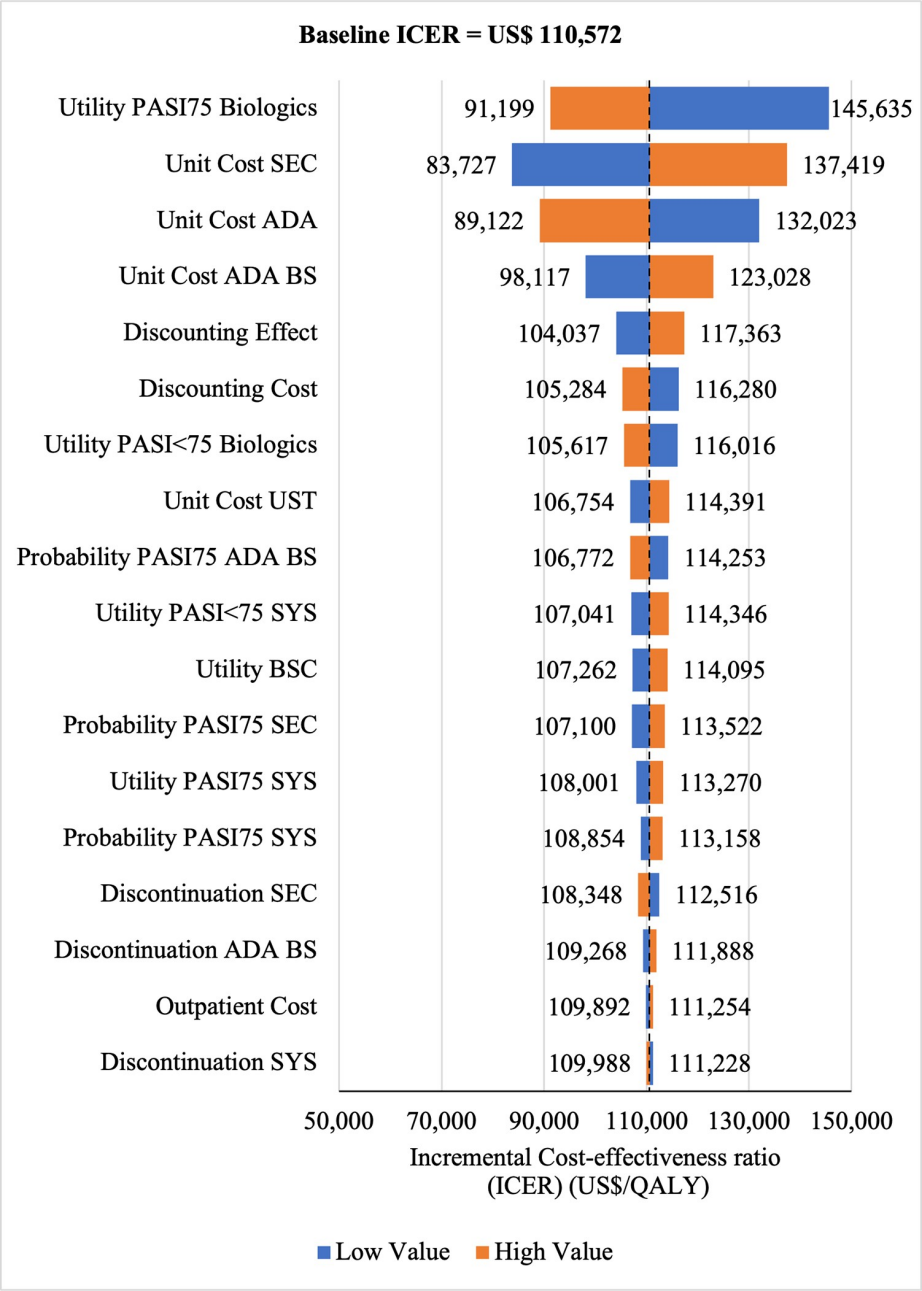
One way sensitivity analysis (biosimilar sequences versus first line SYS).

**Fig 12:**
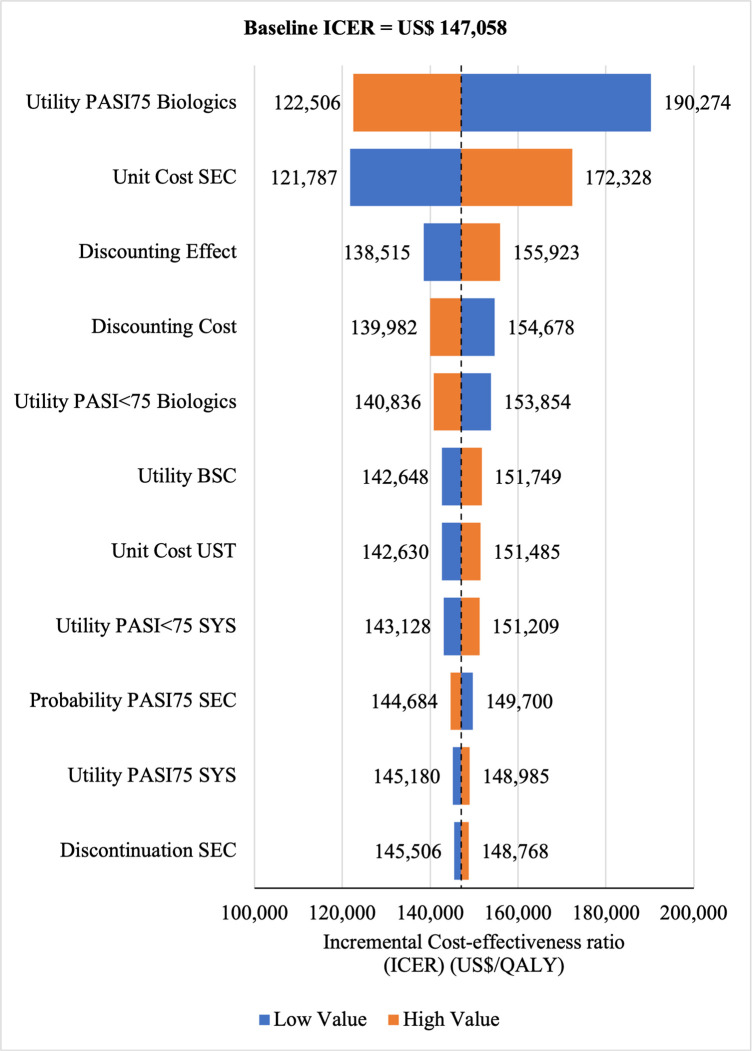
One way sensitivity analysis (reference biologic sequences versus first line PHOTO).

**Fig 13:**
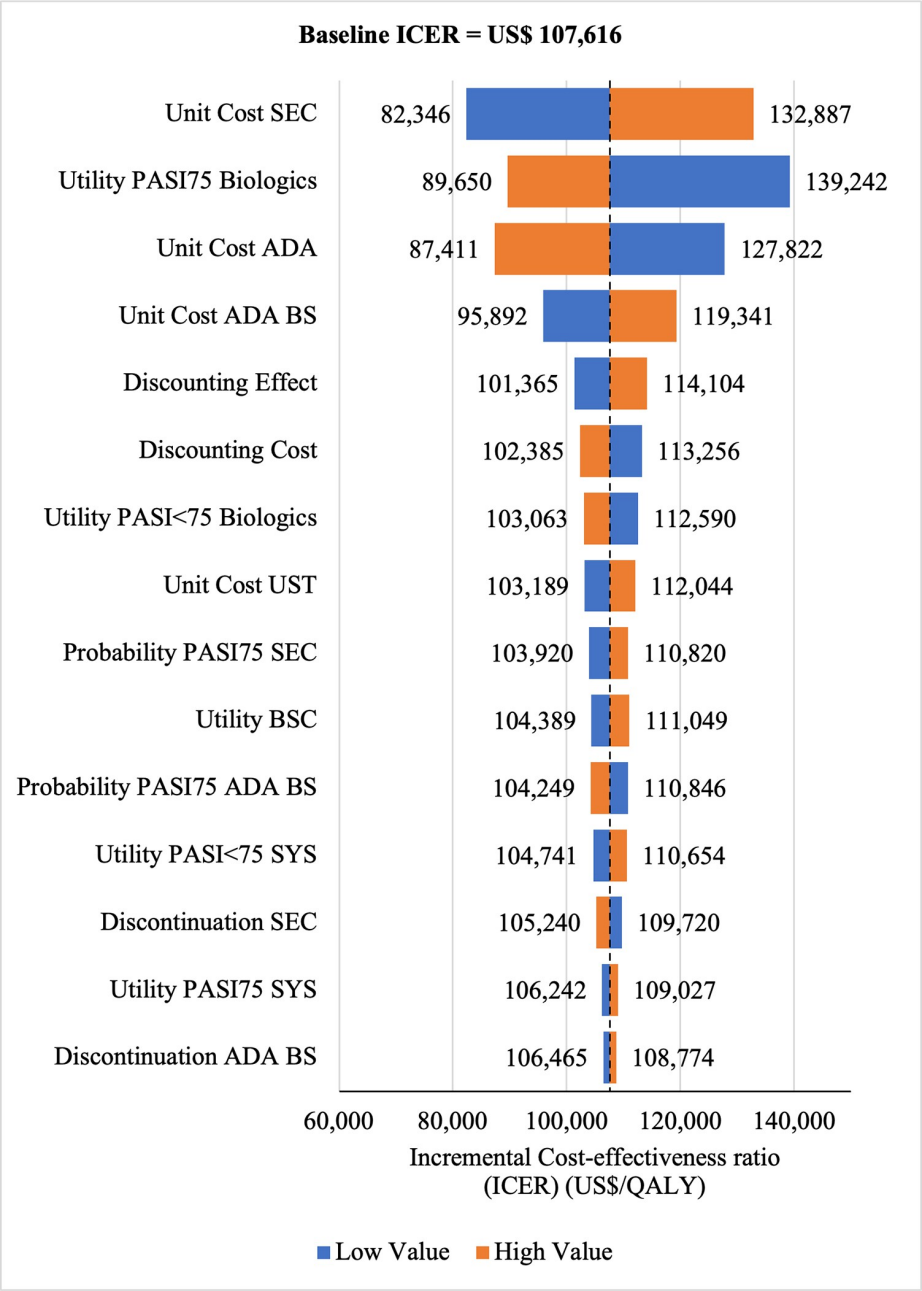
One way sensitivity analysis (biosimilar sequences versus first line PHOTO).

The Tornado diagram showed that utility value had the greatest impact on ICERs for SEC→UST→ADA **(ICERs** range: US$200,823/QALY to US$125,759/QALY) and for SEC→UST→ADA BS (ICERs range: US$145,635/QALY to US$91,199/QALY) vs SYS→UST→ADA as well as for SEC→UST→ADA vs PHOTO→UST→ADA (ICERs range: US$190, 274/QALY to US$122, 506. Meanwhile, ICER had the highest sensitivity to the unit price of SEC for SEC→UST→ADA BS vs PHOTO→UST→ADA (ICERs range: US$82,346/QALY to US$132,887/QALY) (Figs 10–13). Scenario analyses indicated that a reduction of 40% of reference biologic unit costs makes most originator treatment sequences cost effective, ICERs range from US$92,373 to US$111,330 per QALY and ICERs range from US$88,847 to US$106,737 per QALY when compared to SYS→UST→ADA and PHOTO→UST→ADA, respectively (Tables 6 and 7).

**Table 6:** Scenario analysis of the reduction of originator drugs unit costs.

Sequence	20% reduction			30% reduction		
	Total costs (US$)	Total QALYs	ICER (US$/QALY)	Total Cost (US$)	Total QALYs	ICER (US$/QALY)
**SYS→UST→ADA**	124,426	3.30		111,779	3.30	-
SEC→UST→ADA	201,917	3.93	122,424	179,759	3.93	107,398
SEC→ADA→UST	203,033	3.93	124,198	180,737	3.93	108,954
UST→SEC→ADA	209,357	3.94	132,664	186,362	3.94	116,501
UST→ADA→SEC	212,793	3.94	138,254	189,388	3.94	121,423
ADA→SEC→UST	217,217	3.94	144,832	193,256.73	3.94	127,173
ADA→UST→SEC	218,581	3.94	147,183	194,465	3.94	129,256
**PHOTO→UST→ADA**	122,600	3.26		110,228	3.3	-
SEC→UST→ADA	201,917	3.93	117,952	179,759	3.93	103,399
SEC→ADA→UST	203,033	3.93	119,622	180,737	3.93	104,863
UST→SEC→ADA	209,357	3.94	127,645	186,362	3.94	112,016
UST→ADA→SEC	212,793	3.94	132,902	189,388	3.94	116,645
ADA→SEC→UST	217,217	3.94	139,111	193,256	3.94	122,072
ADA→UST→SEC	218,581	3.94	141,317	194,465	3.94	124,027

**Table 7:** Scenario analysis of the reduction of originator drugs unit costs.

Sequence	40% reduction			50% reduction		
	Total costs (US$)	Total QALYs	ICER (US$/QALY)	Total Cost (US$)	Total QALYs	ICER (US$/QALY)
**SYS→UST→ADA**	99,131	3.30	-	86,484.63	3.30	-
SEC→UST→ADA	157,601	3.93	92,373	135,443.89	3.93	77,348
SEC→ADA→UST	158,442	3.93	93,710	136,146.80	3.93	78,466
UST→SEC→ADA	163,368	3.94	100,339	140,374.80	3.94	84,178
UST→ADA→SEC	165,984	3.94	104,594	142,579.96	3.94	87,763
ADA→SEC→UST	169,296	3.94	109,515	145,335.65	3.94	91,857
ADA→UST→SEC	170,350	3.94	111,330	146,235.83	3.94	93,404
**PHOTO→UST→ADA**	97,856	3.3	-	85,485	3.26	**-**
SEC→UST→ADA	157,601	3.93	88,847	135,444	3.93	74,294
SEC→ADA→UST	158,442	3.93	90,104	136,147	3.93	75,346
UST→SEC→ADA	163,368	3.94	96,388	140,375	3.94	80,760
UST→ADA→SEC	165,984	3.94	100,388	142,580	3.94	84,132
ADA→SEC→UST	169,296	3.94	105,034	145,336	3.94	87,996
ADA→UST→SEC	170,350	3.94	106,737	146,236	3.94	89,447

**Fig 14:**
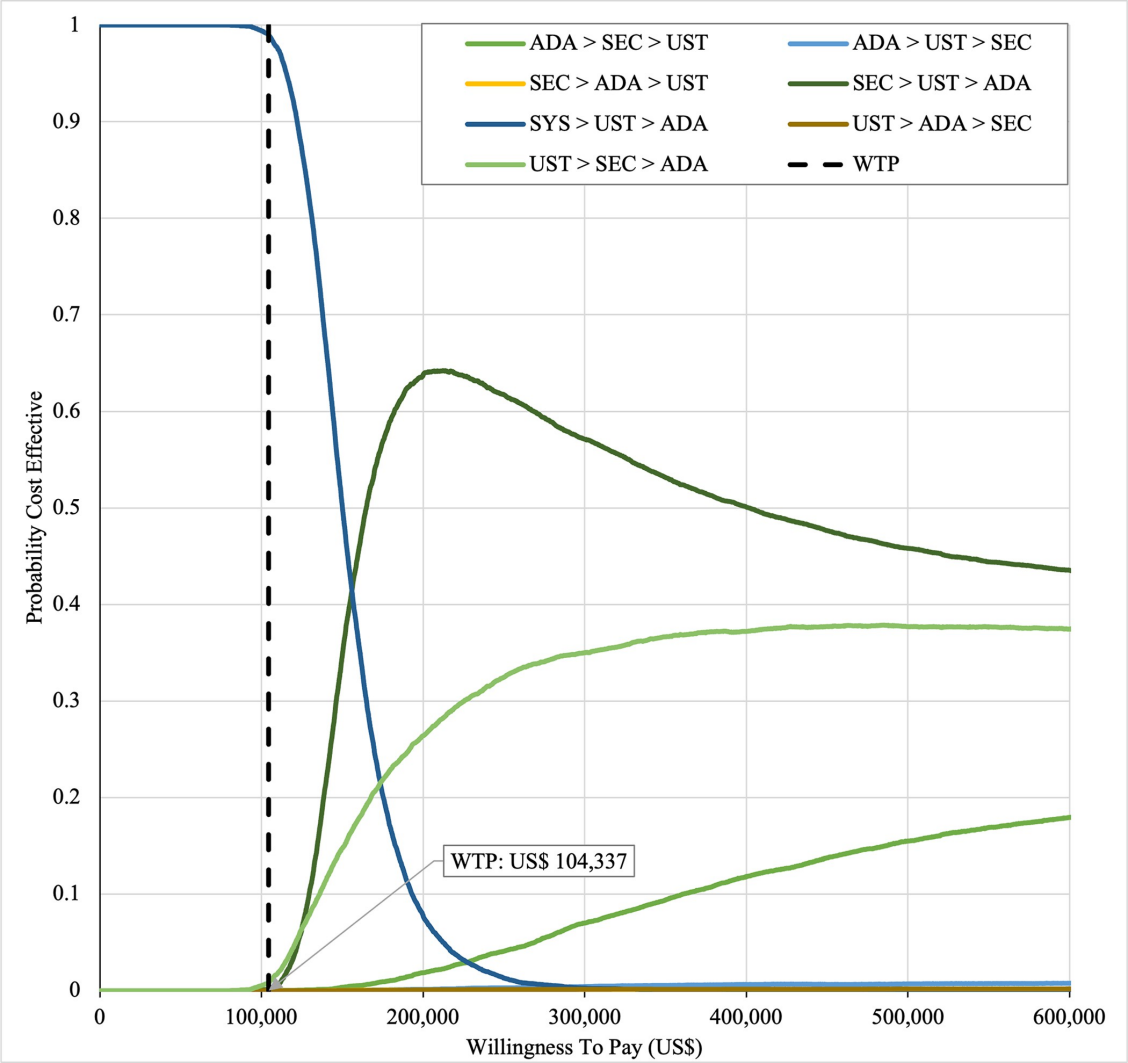
Cost effectiveness acceptability curve (reference biologic sequences versus first line SYS).

**Fig 15:**
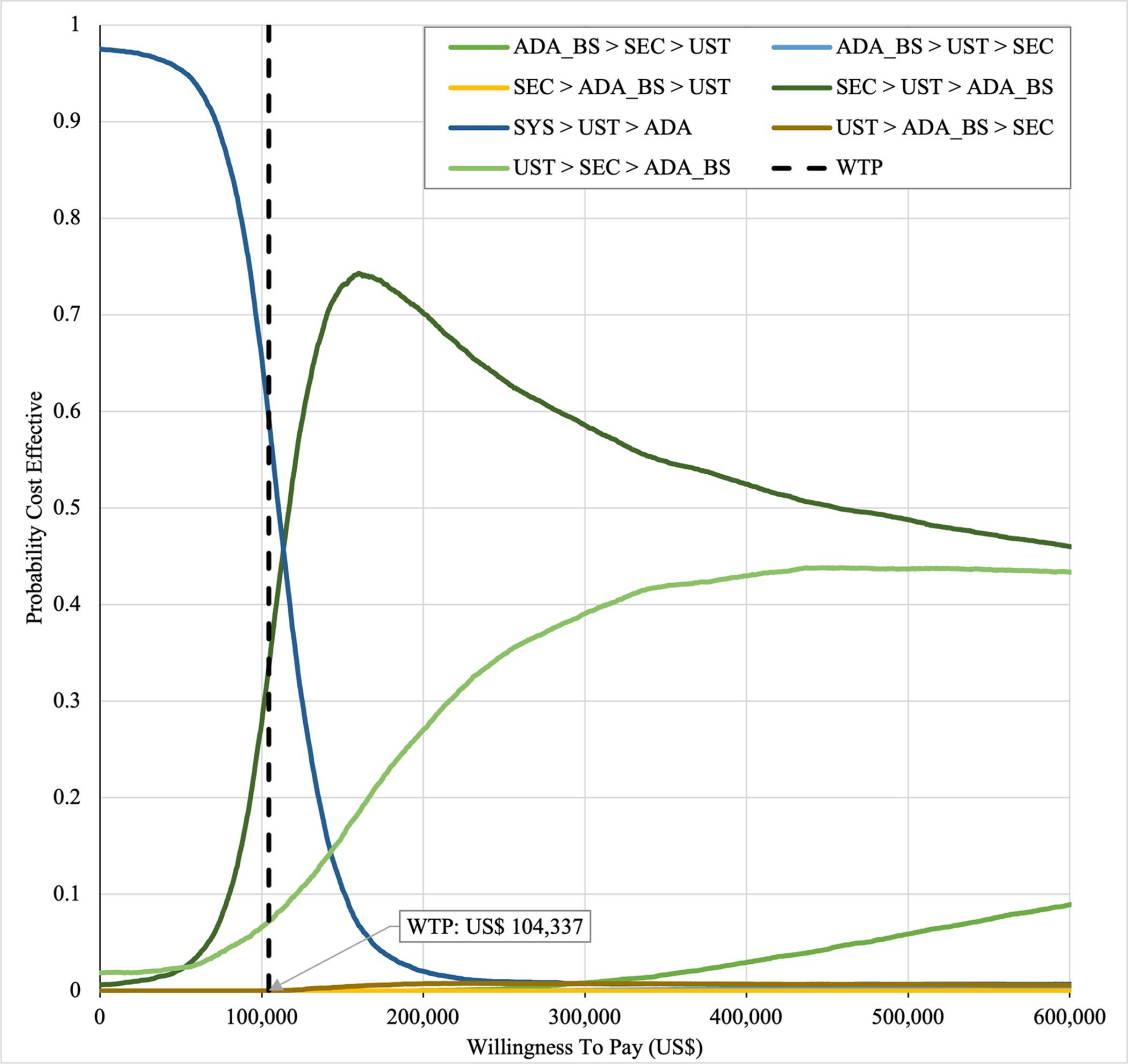
Cost effectiveness acceptability curve (biosimilar sequences versus first line SYS).

**Fig 16:**
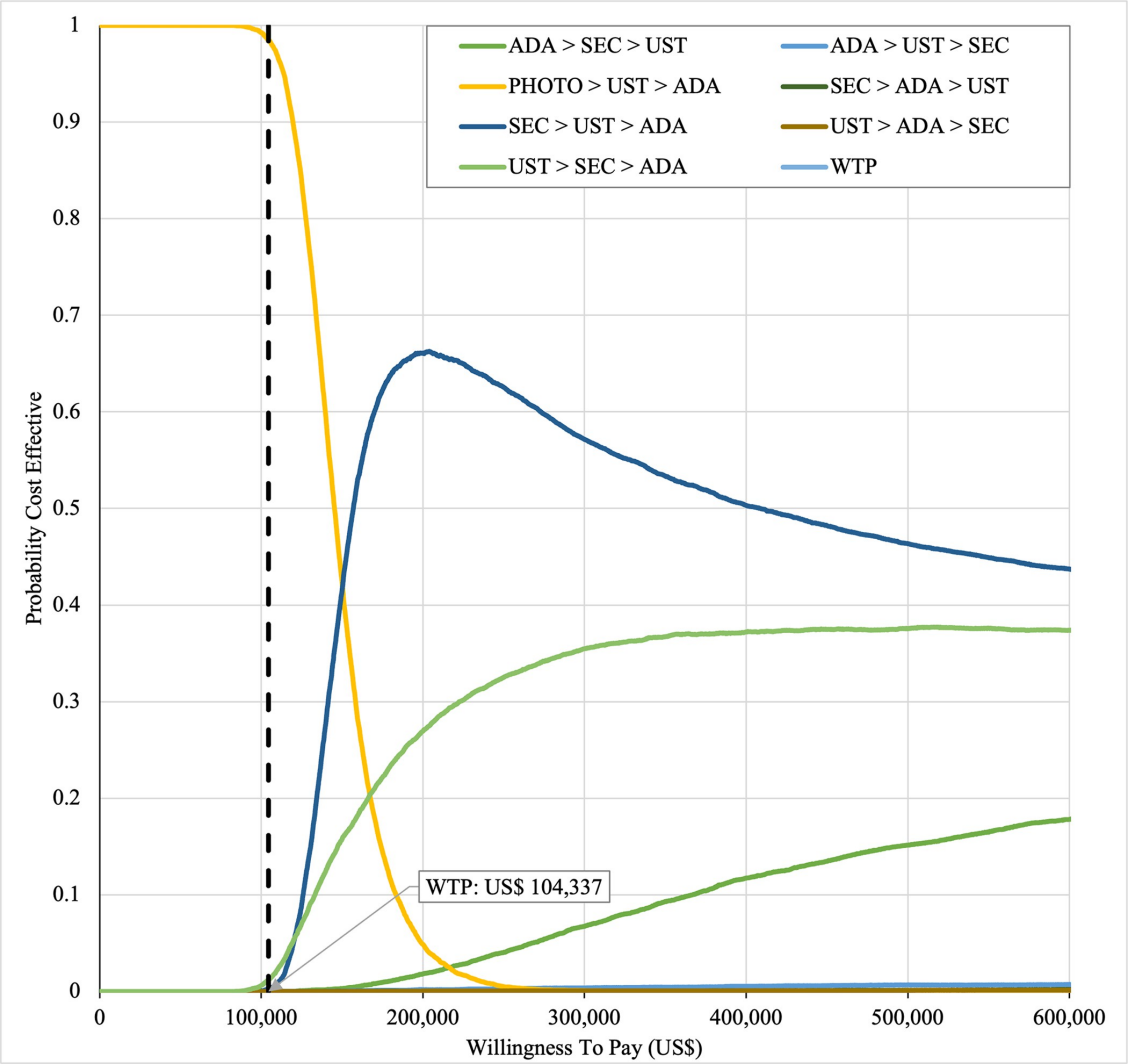
Expected loss curves (reference biologic sequences versus first line SYS).

**Fig 17:**
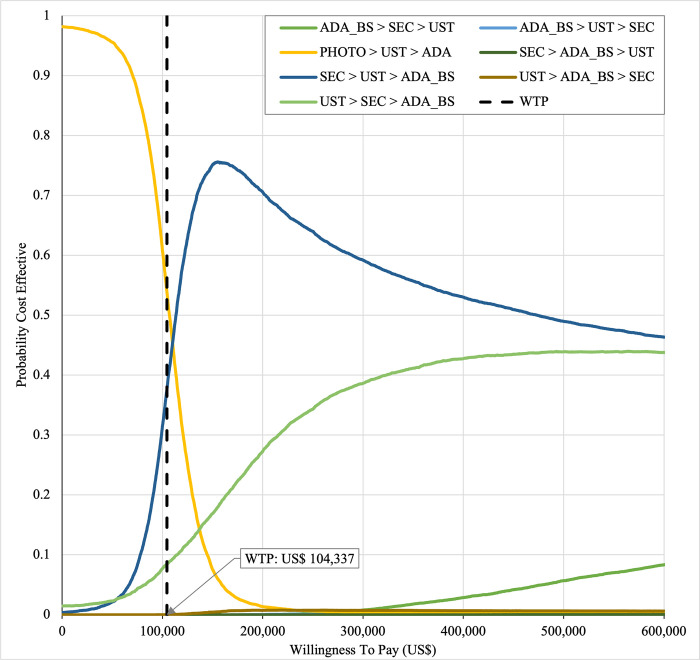
Expected loss curves (biosimilar sequences versus first line SYS).

SYS→UST→ADA had the highest probability of being the most cost-effective treatment at national WTP threshold of US$104,337/QALY in both first and second set analyses. The probability of being cost effective was highest for SEC→UST→ADA only at WTP value greater than US$145,949/QALY and SEC→UST→ADA BS only at greater than US$115,000/QALY (Figs 14 and 15). The comparator sequence also the lowest expected loss (maximum loss; US$9,335) at WTP less than US$145,886/QALY. SEC→UST→ADA had the lowest expected loss (maximum loss; US$10,466) at WTP between US$145,949 to US$320,189 per QALY. SEC→UST→ADA BS had the lowest expected loss (maximum loss; US$9,746) at WTP between US$115,000 to US$306,265per QALY (Figs 16 and 17).

**Fig 18:**
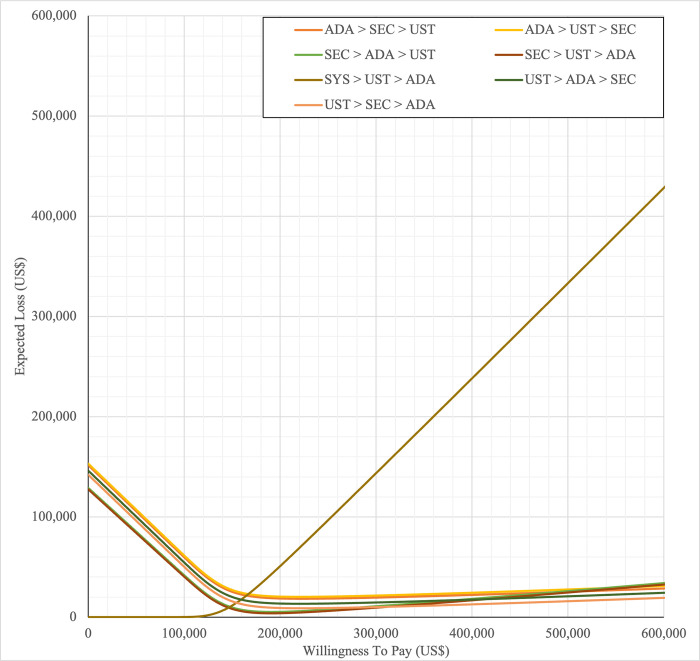
Cost effectiveness acceptability curve (reference biologic sequences versus first line PHOTO).

**Fig 19:**
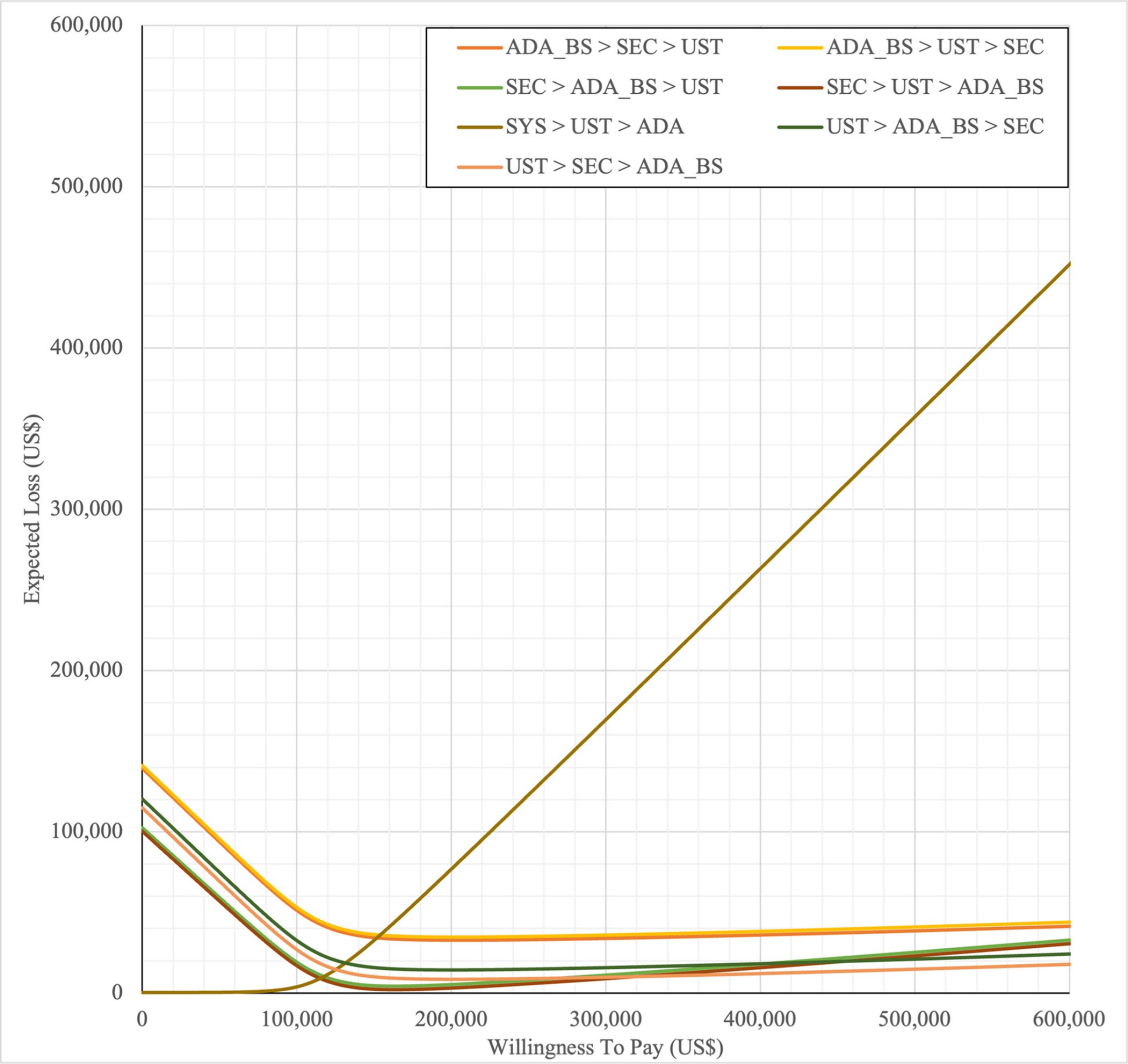
Cost effectiveness acceptability curve (biosimilar sequences versus first line PHOTO).

**Fig 20:**
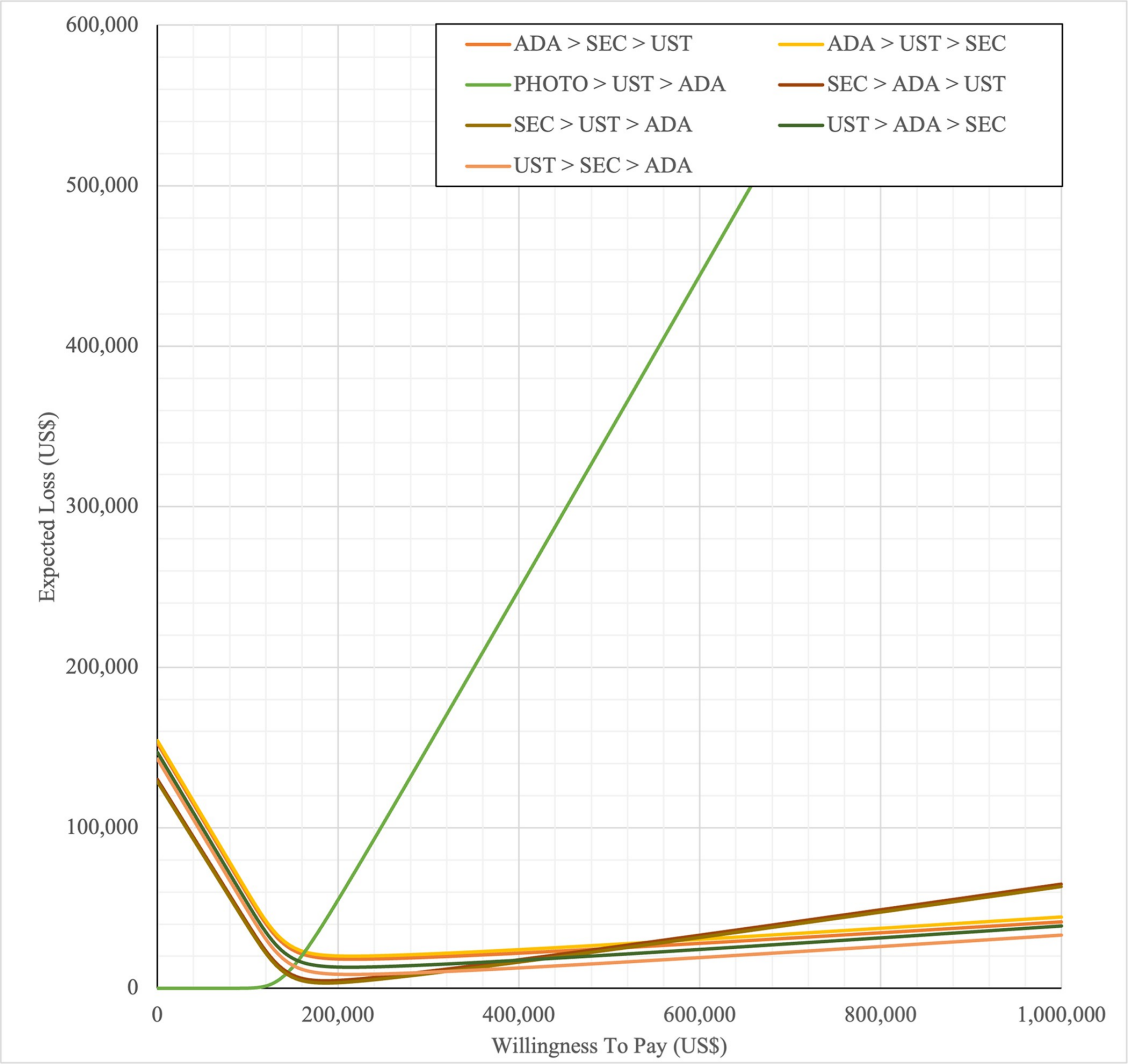
Expected loss curves (reference biologic references versus first line PHOTO).

**Fig 21:**
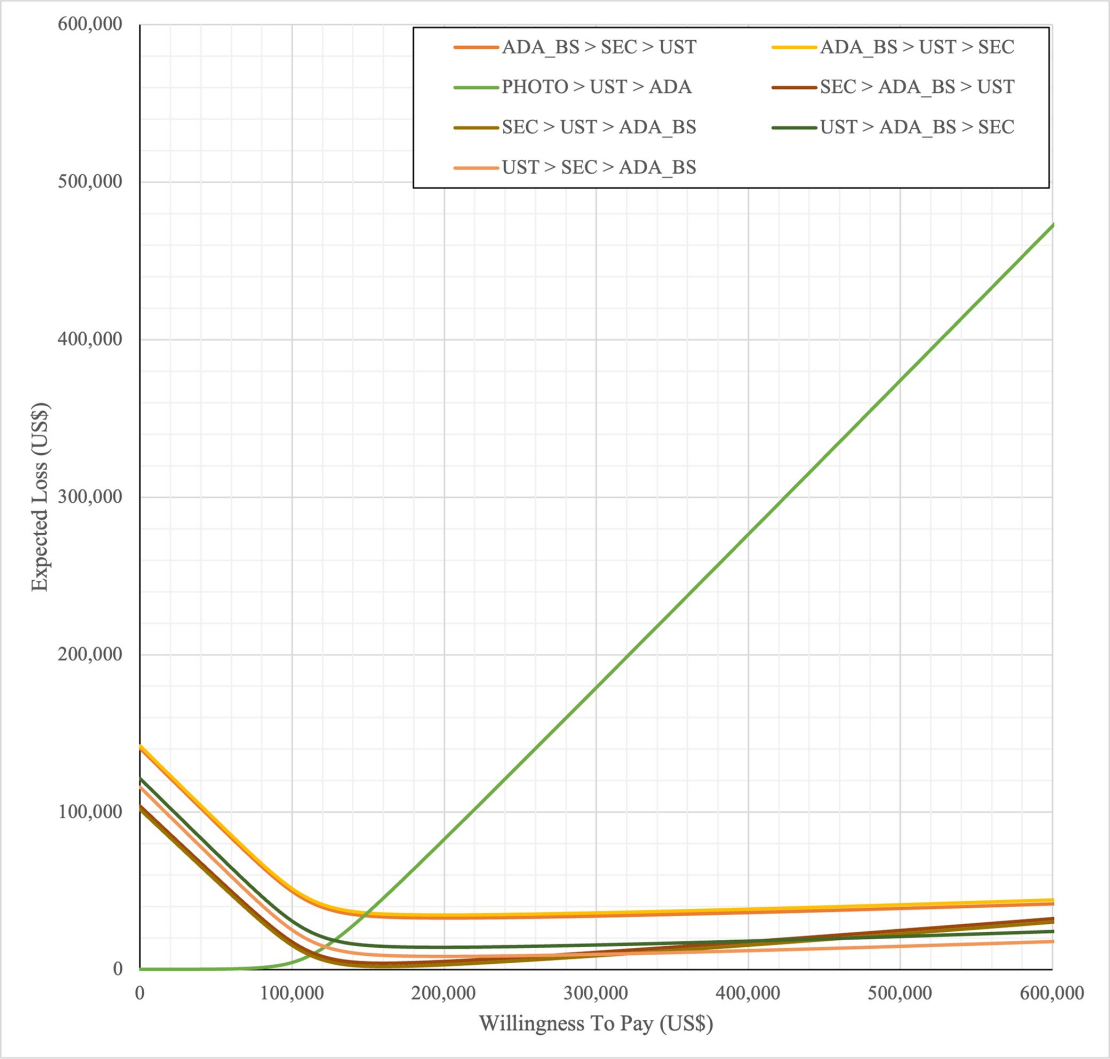
Expected loss curves (biosimilar sequences versus first line PHOTO).

Similarly, PHOTO→UST→ADA sequence had highest probability of being cost-effective at national CET in both set analyses. The probability of being cost effective was highest for SEC→UST→ADA only at WTP greater than US$142,151 per QALY and SEC→UST→ADA BS only at WTP greater than US$112,721 (Figs 18 and 19). The comparator sequence also demonstrated the lowest expected loss (maximum loss; US$8,929) at WTP value less than US$142,088. At WTP greater than US$142,151, SEC→UST→ADA had the lowest expected loss (maximum loss; US$10,209). The comparator sequence also showed the lowest expected loss at WTP less than US$112,658 (maximum loss; US$9,308). At WTP between US$112,784 to US$319,936, SEC→UST→ADA BS had the lowest expected loss (maximum loss; US$10,067) (Figs 20 and 21).

## Discussion

This is the first study to investigate the costs and effects of biologics and introduction of biosimilar for moderate to severe psoriasis in Malaysia. While there have been some economic evaluation studies on psoriasis treatments in Southeast Asia, none of them have estimated the costs and effectiveness of biologic sequential treatments[19,63,64]. Consequently, there is currently unclear evidence on the most cost-effective treatment sequence in this region. This evaluation utilises the abundant comparative evidence available in this field to determine the most cost-effective sequence of treatments in Malaysia. This was accomplished by determining the cost-effectiveness of treatment sequencing consisting of a reference biologic and then increasing the cost-effectiveness of this sequence by substituting a biosimilar for the adalimumab. Biosimilar adalimumab (brand name Amgevita) has been approved for psoriasis in Malaysia [24], however, there is no information available regarding its utilization in the management of psoriasis.

Results from this analysis suggests that introduction of ADA BS to treatment sequences were cost saving strategies compared to first line SYS and first line PHOTO comparators as it reduced the costs of treatment without significant loss of effectiveness (Tables 4 and 5). The estimated cost saving associated with replacing ADA with its biosimilar was estimated to be US$11,420 to US$26,522 and per person treated compared to both comparators. This finding aligns with previous studies that have assessed the cost-effectiveness of biosimilars for psoriasis as well as other diseases [23,65,66]. The consistency across these studies emphasizes the significance of this input for decision-makers, suggesting the potential benefit of introducing biosimilars earlier in the management of psoriasis. Such an approach could enhance patient accessibility to optimal treatments for moderate to severe psoriasis. As noted by Asmah et al. [8], a significant proportion of psoriasis patients in Malaysia currently receive suboptimal treatments, further emphasizing the need for interventions that can improve the overall management of the condition. First-line SEC sequences were the most cost-effective of all cost-efficient sequences. This supported the recommendation made by Malaysian dermatologists that SEC should be considered as a first-line option among biologic therapies in Malaysia [8]. Given the highly subsidized nature of Malaysian healthcare system, the adoption of cost-saving strategies holds particular importance to ensure the efficient allocation of resources and the sustainability of the healthcare system [67].

The analysis’s robustness was confirmed through sensitivity analyses. OWSA revealed that the unit cost of SEC was the most sensitive parameter. The studies in Brazil [36] and USA [61] reported similar results, in which that cost of biologics had the greatest impact on ICERs. An important finding from scenario analyses were the identification of threshold values. Specifically, reducing the acquisition cost of SEC by 40.0% would render the treatment sequences consisting of biosimilar and most of its reference drugs sequences cost-effective. These data can be of great utility to stakeholders in determining specific pricing strategies in Malaysian setting. Although few economic evaluation studies in Malaysia discussed CET values, decisions were made without using a transparent and solid threshold value. The funding and reimbursement process in Malaysia use one to three times GDP per capita, as per the recommendation of the WHO for interpreting cost-effectiveness findings [68]. In the absence of specific CET in Malaysia to assess the value for money of healthcare interventions, understanding the ICERs derived from existing economic evaluation studies can provide guidance in establishing an acceptable threshold [69]. Typically, the disease severity represents the average quantity of health lost by a population affected by a specific disease. Adopting variable cost-effectiveness thresholds based on severity, as opposed to a fixed threshold, is believed to promote a more equitable distribution of resources, despite the possibility of a decline in the aggregate health of the society [70]. Moderate-to-severe psoriasis has a profound impact on patients, and associated with various comorbidities, including psoriatic arthritis, obesity, hypertension, cancers, metabolic syndrome, and cardiovascular disease [71–73]. Taking account to these burdens, the threshold ranges from US$107,616 to US$183,036/QALY could be considered for moderate to severe psoriasis condition in Malaysia. According to Ku Abd Rahim et al. [69], none of the economic evaluation studies have reported expected loss associated with choosing suboptimal strategy. To provide additional insights, the current analysis quantified the anticipated loss associated with choosing sub-optimal strategy across a range of WTP thresholds.

In incremental analysis, all treatment sequences were compared to common comparators, which were (SYS➔UST➔ADA and PHOTO➔UST➔ADA), and this was designed to respond to the current decision problem in the management of psoriasis in Malaysia. The current Malaysian guideline on psoriasis management recommends systemic therapy and phototherapy as first-line or standard treatments for moderate to severe psoriasis [15]. Malaysian dermatologists believe that biologic therapy should be initiated earlier in the management of psoriasis than currently recommended. Among all biologics, they advocate for secukinumab to be used as a first-line treatment [8]. However, this proposal is based solely on the clinical efficacy and safety profiles of biologics, without evidence of cost considerations [8]. Malaysia has begun incorporating pharmacoeconomic evidence into regulatory assessments of new drugs that necessitate economic analysis. This initiative aims to provide stakeholders, health systems, and governments with comprehensive economic information to inform policy development [26]. Therefore, information regarding the additional costs required to achieve additional benefits of treatments compared to most currently used treatments provides valuable insights to justify resource allocation for the improvement of the management of psoriasis in Malaysia. [15,74–76] With a total of nine biologics being used for moderate to severe psoriasis in Malaysia [16], there exist a hypothetical combination of 504 different three-biologic treatment sequences. However, including all possible permutations in the analysis was not feasible in this analysis. Therefore, the number of permutations was narrowed down by focusing on the most prescribed biologics for psoriasis patients, consistent with Baker et al. [23] in the UK. The choice of treatment sequence for psoriasis has become more challenging due to the continuous development of biologics with different modes of action and the introduction of new biosimilars [23]. To address this complexity, the model in this study analyzed three lines of targeted reference biologics and biosimilar. By considering three lines of biologic treatment, this analysis provides insights into how the expanding practice of switching therapies has increased the range of options for patients who do not respond to one or two lines of treatment. This approach accounts for the evolving landscape of biologic therapies and reflects the current switching practices in the management of psoriasis. Among all economic analyses evaluating the cost and efficacy of psoriasis treatments [77], few studies evaluate three-line sequential biologic therapy. These studies have utilized different interventions, comparators, time horizons, and sets of permutations, drawing from previous analyses. As a result, the findings regarding the most cost-effective treatment sequence have been varied and inconclusive [23,25,37,61].

In Malaysia, one of the common obstacles to conduct health economic evaluations is difficulties in gaining access to local data [69]. The MPR was established to provides information such as demographic data, clinical data, quality of life evaluation, and treatments used. Unfortunately, PASI data was not reported in the registry [16], rendering economic evaluation using local data difficult. Considering these data limitations, health economic modeling can play a crucial role in addressing this issue. Consequently, the current analysis provided important information to bridge the information gaps and fill the existing loopholes in health economics research in Malaysia. Because psoriasis is a chronic disease, its treatment lasts for lifetime, hence, a lifetime horizon was adopted, consistent with prior psoriasis studies [31–33]. One of the key strengths of this analysis include reliable input source to build the model. In line with the International Society for Pharmacoeconomics and Outcomes Research and the Society for Medical Decision-Making Modeling Task Force [78] recommendation, the transition probabilities and intervention effects in this analysis were derived from the most pertinent data sources. In particular, the efficacy data utilised in this analysis were taken from a large NMA based on RCTs of biologics for the moderate to severe psoriasis. The NMA is considered the most reliable source for deriving accurate transition probabilities when comparing multiple interventions [79].

Nevertheless, this analysis subjected to limitations, as it relies on certain assumptions and estimates based on the available evidence. An important consideration is that the efficacy parameters obtained from the NMA may be influenced by variations in trial protocols and patient characteristics, which may impact the treatment’s efficacy. Although the adjustment made for the response in the reference arm within the NMA helps to mitigate potential confounding due to trial heterogeneity, it does not completely eradicate the effect on the generalizability of the results to the Malaysian population. Additionally, because of a lack of available published data on utilities specific to Malaysia, the input for this study was derived from PASI scores, which were estimated from the general population in the UK. Notably, these utility values were calculated using UK tariffs, which may not accurately depict the utility values of the Malaysian patient population. Due to practical limitations, the analysis included only a limited number of treatment options, as it is not feasible to consider every possible treatment sequence. The selection of these specific treatments was based on the current prescribed biologic treatment for psoriasis patients in Malaysia [16], ensuring that they represent a relevant decision problem. It is important to note that the official guidelines for the management of psoriasis in Malaysia provide limited recommendations regarding the choice of biologic treatment sequences for psoriasis. Additionally, robust evidence supporting the recommendation of specific biologic switching at a national level in Malaysia is lacking.

The treatment sequences analyzed in the study showed minimal differences in total QALYs. One possible explanation for this could be the slight variations in discontinuation rates between the different treatments. Should the difference efficacy of biologics be greater, the QALY difference between treatments would likely be greater [61]. The similar utility findings between all treatment arms are common for modelling evaluation treatment sequences as seen in previous studies [23,25]. Therefore, biologic acquisition cost and dosage frequency play a significant role in determining cost-effectiveness in this study. Although SEC had a higher acquisition cost than adalimumab, the greater dosage frequency of ADA makes SEC the most cost-effective first-line treatment in Malaysia. In this analysis, patients who did not achieve a response at the end of the induction period were switched to second-line or third-line treatments, or to BSC. In real-world clinical practice, clinicians may consider dose escalation as an alternative option [15]. Dose escalation could have a substantial impact on the results of the analysis, particularly considering that drug costs were a significant factor in the comparative cost-effectiveness [61] However, the current study did not incorporate this impact due to limitations in available data. It is crucial to note that this economic evaluation solely offer recommendations from an economic standpoint. Dermatologists, on the other hand, need to consider various other factors when selecting a biologic treatment for their patients. These factors include patient comorbidities, compliance with the treatment regimen, and dosing requirements. For instance, in patients with psoriatic arthritis or concurrent cardiovascular risk factors, opting for TNF-α inhibitors may enable the management of multiple disease processes concurrently. Conversely, the use of TNF-α inhibitors is contraindicated in patients with a history of malignancy or multiple sclerosis. Hence, dermatologists must take into account these additional considerations beyond cost-effectiveness when making treatment decisions for their patients [25].

## Conclusion

This analysis showed that adopting biosimilar into the treatment sequences could achieve cost savings ranging from 4.3% to 10.8%. This could be one solution to improve patient access to optimal treatments for moderate to severe psoriasis in Malaysia. It should be noted that a reference biologic treatment sequence would be cost-effective if Malaysia has a local threshold that allows for a higher value, potentially up to US$184,000/QALY for severe disease conditions, considering the burden of psoriasis to the patients. The implementation of treatment sequencing offers a more realistic representation of the decision problem and its health economic implications. This approach takes into account treatment guidelines and clinical practice, which are particularly relevant considering the long-life expectancy of patients and the probability of receiving multiple biologic treatments over their lifetime.
